# Integrated spectroscopic and morphological analyses reveal cellular shifts in gene-silenced melanoma CSCs

**DOI:** 10.1038/s41598-025-17155-2

**Published:** 2025-08-25

**Authors:** Berrin Ozdil, Günnur Güler, Evren Ataman, Huseyin Aktug

**Affiliations:** 1 Department of Histology and Embryology, Faculty of Medicine, Ege University, Izmir, 35100 Turkey; 2 Department of Histology and Embryology, Faculty of Medicine, Suleyman Demirel University, Isparta, 32260 Turkey; 3 Department of Physics, Izmir Institute of Technology, Izmir, 35430 Turkey

**Keywords:** Malignant melanoma, Cancer stem cell, Cytoskeleton, ATR-FTIR, SEM/EDS/XPS, Biophysical chemistry, Optical physics, Optical techniques, Skin cancer, Cancer stem cells, Skin cancer

## Abstract

Intratumoral heterogeneity remains a major barrier to durable cancer therapies, largely driven by the persistence of cancer stem cells (CSCs). In this study, we employed an integrated, multi-scale approach to investigate how melanoma CSCs respond to siRNA-mediated silencing of three key regulatory genes: KLF4, SHH, and HIF1α. Using a combination of morphological, molecular, spectroscopic, and elemental analyses, we explored structural and biochemical consequences of gene knockdown. Gene silencing resulted in significant changes in cell shape and size, reduced F-actin organization, and decreased PFN1 expression, indicating a loss of stem-like properties. ATR-FTIR spectroscopy revealed shifts in biomolecular composition, notably a reduction in amide III intensity and an increase in lipid ester content. SEM-EDS point-based elemental analysis revealed relative differences in carbon and nitrogen levels between selected central and peripheral regions of silenced and control cells, at the micron-scale working depth, reflecting broader elemental distribution trends rather than precise subcellular compartmentalization. XPS analysis further confirmed these differences, providing additional insights into the elemental composition of the cellular surface. The integration of FTIR spectroscopy into this study highlights the potential of infrared spectroscopy as a powerful tool in cancer research. These findings demonstrate that targeting critical regulatory pathways induces cytoskeletal and biochemical remodelling in melanoma CSCs, offering a multi-dimensional perspective on cellular plasticity.

## Introduction

Malignant melanoma, an exceedingly aggressive cutaneous tumor originating from melanocytes, exhibits a multifaceted genetic landscape influenced by a combination of genetic and environmental factors^1–4^. The heightened aggressiveness of malignant melanoma is closely linked to the presence and activity of cancer stem cells (CSCs), a characteristic shared with various other cancer types. The presence of stem cells within malignant melanoma, along with their involvement in its initiation and progression, is strongly indicated by the observation of tumor heterogeneity, the presence of undifferentiated molecular markers, and an increased resemblance of melanoma subtypes to embryonic-like developmental states^5,6^.

The genes KLF4, SHH, and HIF1α were selected based on their distinct yet functionally intertwined roles in CSC maintenance. KLF4 contributes to stemness and resistance to differentiation; SHH signaling is essential for self-renewal and tumor initiation; and HIF1α mediates cellular adaptation to hypoxic stress. These regulatory factors are frequently dysregulated in melanoma and cooperate to sustain CSC viability, invasiveness, and therapeutic resistance. Previous studies, including our earlier work^7^have demonstrated that silencing these genes alters miRNA expression profiles and disrupts CSC-associated features in melanoma.

The role of CSCs in malignant melanoma further underscores the intricate connection between melanoma and the cytoskeleton. Emerging evidence suggests that CSCs contribute to tumor initiation, progression, and therapy resistance in melanoma. These cells often display distinct cytoskeletal dynamics that enable their invasive and metastatic behavior. The regulation of cytoskeletal elements, such as actin filaments, microtubules, and associated proteins, is crucial for maintaining the stemness and plasticity of CSCs in melanoma. Disrupting these cytoskeletal dynamics may represent a promising avenue for targeting CSCs and preventing tumor recurrence and metastasis in melanoma patients^8–10^. Cytoskeletal elements, including microtubules and actin filaments, are involved in key processes that are critical for melanoma cell invasion and metastasis^11,12^. Several studies have highlighted the importance of cytoskeletal dynamics in melanoma, with cytoskeletal alterations contributing to the acquisition of invasive phenotypes by melanoma cells. Understanding the complex association between melanoma and the cytoskeleton is vital for the development of targeted therapeutic strategies aimed at disrupting these processes and improving patient outcomes.

Given the cytoskeletal remodelling observed in melanoma cells, advanced imaging techniques are essential to characterize structural changes at high resolution. The scanning electron microscope (SEM) plays a pivotal role in elucidating the intricate relationship between the cytoskeleton and cancer biology, particularly in malignant melanoma and CSCs. SEM is a powerful imaging technique that allows high-resolution, three-dimensional visualization of cellular morphology. In the context of malignant melanoma and CSCs, SEM can reveal distinctive cellular dynamics, including morphological features such as increased filopodia and lamellipodia formation, which are associated with invasive and metastatic potential^13^. This approach also enables detailed examination of structural changes that may influence CSC behavior within melanoma tumors. Furthermore, SEM can be coupled with energy-dispersive X-ray spectroscopy (EDS) to obtain complementary information on the elemental composition of cellular structures^14,15^. Such combined analyses can provide valuable insights into how malignant melanoma and CSCs are affected by altered gene expression or therapeutic interventions.

Fourier Transform Infrared (FTIR) spectroscopy has become a widely used technique in molecular biology research in the past 20 years and is rapidly gaining popularity in cancer biology. FTIR spectroscopy provides valuable chemical insights and has been increasingly used to classify biological samples, including cancer tissues, based on molecular composition^16^. This research aims to gain insights into the molecular characteristics and spectral signatures of melanoma cells, which can aid in understanding their metastatic behaviour and, ultimately, improve diagnostic and treatment strategies for this aggressive form of cancer^17–19^. To our knowledge, this is the first study to apply ATR-FTIR spectroscopy to malignant melanoma CSCs, highlighting its potential for assessing metastatic potential and identifying novel biomarkers. Analyzing CSCs with FTIR could offer molecular-level insights into their metastatic potential, ultimately aiding in prognostic assessment and targeted therapy development^20^. FTIR spectroscopy has been presented as a technique that can be used both as an experimental characterization technique in cancer biology^21–23^ and as a method that can reveal the differences between stem cell biology, somatic cells, cancer cells, and embryonic cells^23^.

X-ray Photoelectron Spectroscopy (XPS) enables quantitative surface analysis of cellular samples, detecting atomic composition at nanometric depth (20–100 Å)^24^. Despite being traditionally used in materials science^25^its application to biological systems remains limited^14,15,26,27^. In this study, XPS was employed to examine the elemental alterations in CSCs following gene silencing, complementing molecular and morphological findings.

In this study, we aimed to investigate how silencing of the KLF4, SHH, and HIF1α genes affects the cytoskeletal structure and biochemical characteristics of melanoma CSCs. To achieve this, we employed an integrated experimental design combining RT-PCR (gene expression), F-actin immunostaining and SEM (morphological analysis), ATR-FTIR spectroscopy (biochemical profiling), and SEM-EDS/XPS (elemental surface composition). This multi-angled approach allowed us to explore how gene silencing influences CSC structure and function at the molecular, cellular, and surface levels.

## Results

Here, we elucidate the outcomes of our comprehensive investigations into malignant melanoma CSCs, which encompassed a multifaceted exploration of molecular, structural, and elemental analyses. These findings shed light on the intricate interplay between gene expression, cellular morphology, and elemental distribution within the context of CSC biology.

### Profilin gene response to KLF4, SHH, and HIF1α Silencing

The expression levels of PFN1 and PFN2 genes were evaluated according to fold change results (2^(-ΔΔCt)) (SI Fig. 1). The genes exhibited comparable patterns between the CD133 + and CD133- cell groups (*p* > 0.05). However, in the siRNA-treated groups, the PFN2 gene showed similar expression levels to the CD133 + cell group (*p* > 0.05), while there was a reduction in the expression levels of the PFN2 gene in CD133+/HIF1α- (*p* = 0.04).

### Dynamic changes in F-actin intensity KLF4, SHH, and HIF1α silencing

**Fig. 1:**
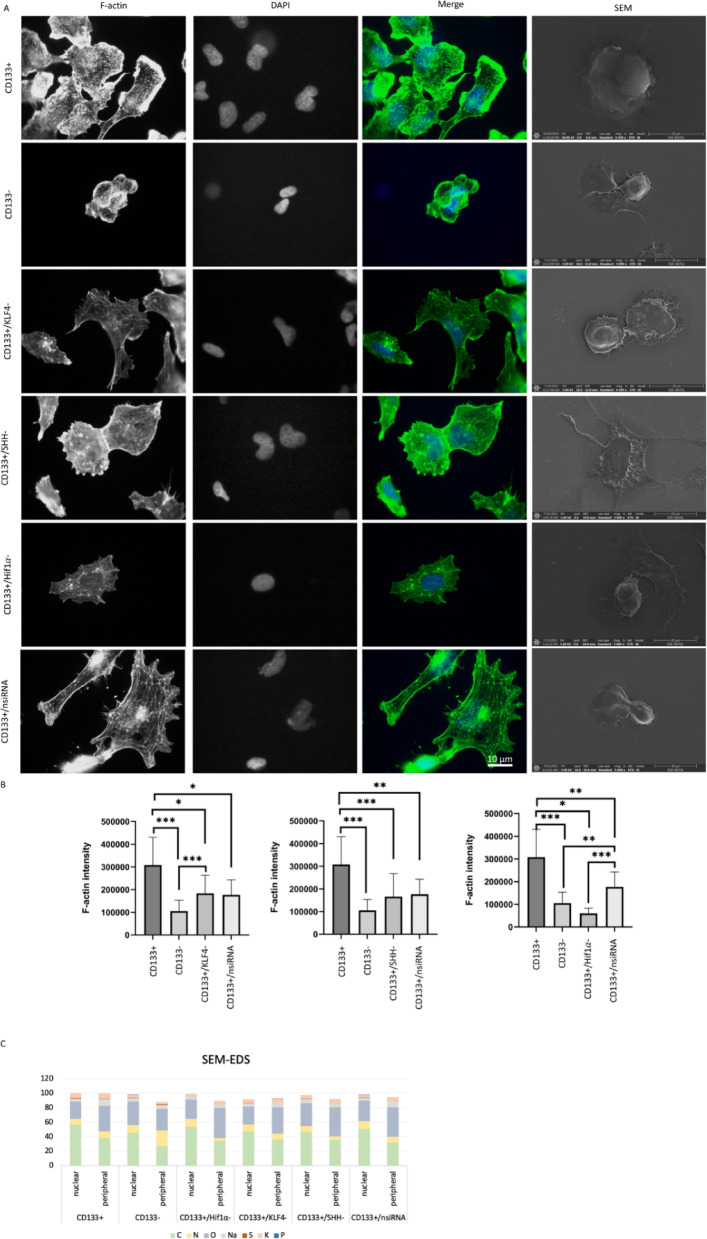
Actin cytoskeletal organization, morphological changes, and elemental composition analysis of cell groups. F-actin levels of cell groups according to CD133 + and CD133- cell groups after siRNA application. Scale bar: 10 μm. Images of the cell groups 100 × (**A**). The green color represents F-actin and blue for DAPI in merge images. The statistical comparison was shown in (**B**) for CD133+/KLF4-, CD133+/SHH-, and CD133+/ HIF1α-. *p < 0.05, **p < 0.01, ***p < 0.001. The rightmost column displays scanning electron microscopy (SEM) images showing morphological differences between the groups. Scale bar: 20 μm. SEM-EDS analysis showing elemental composition ratio in central and peripheral regions of CD133 + and CD133 − cells, as well as CD133 + cells subjected to KLF4, SHH, and Hif1α silencing (**C**).

For the cytoskeletal examination, F-actin staining was assessed. The CD133 + cell group is statistically distinct from the CD133- cell group (*p* < 0.001), and the CD133 + cell group exhibits higher F-actin intensity (Fig. 1A-B). The CD133+/KLF4- cell group is statistically different from both the CD133 + and CD133- cell groups (*p* < 0.05 and *p* < 0.001). Furthermore, the CD133+/KLF4- cell group demonstrates lower expression levels compared to the CD133 + cell group but higher expression levels compared to the CD133- cell group. The CD133+/SHH- and CD133+/HIF1α cell groups exhibit lower expression levels compared to the CD133 + cell group (*p* < 0.001 and *p* < 0.05). No significant difference was observed between the CD133- and CD133+/HIF1α cell groups. Following KLF4, SHH, and HIF1α siRNA treatments, a reduction in F-actin levels was spotted.

### Scanning electron microscopy coupled with EDS for characterization of gene silencing

While using SEM images for comparing cell morphologies (Fig. 1), signals from cells were compared based on elemental percentages in the peripheral and central data points.

In addition to comparing cell groups, SEM-EDS analysis was conducted separately for the Matrigel due to the culturing of cells on its surface. The Matrigel composition revealed the presence of carbon, nitrogen, oxygen, sodium, sulfur, and potassium. In the cell-based analysis, carbon content was found to be the highest, while in the Matrigel, the highest percentages were attributed to oxygen and sodium elements (SI Table 1).

The cell groups were evaluated both centrally and near the cell periphery (Table 1).

Elemental analysis revealed the presence of carbon, nitrogen, oxygen, sodium, sulfur, and potassium in the CD133 + cell group, with carbon being the most abundant element. Notably, carbon content decreased towards the peripheral region (Fig. 1, SI Table 2). The CD133- cell group exhibited a similar elemental composition, with the addition of phosphorus (Fig. 1, SI Table 3). While carbon remained the dominant element, its percentage was lower in the central region compared to CD133 + cells. Additionally, phosphorus was detected in the central region of CD133- group, distinguishing it from the CD133 + group.

The CD133+/nsiRNA, CD133+/KLF4-, CD133+/SHH-, and CD133+/HIF1α- cell groups all contained carbon, nitrogen, oxygen, sodium, phosphorus, sulfur, and potassium, except for CD133+/HIF1α- and CD133+/SHH-, where phosphorus was absent (Fig. 1, SI Tables 4, 5, 6 and 7). Across all groups, carbon exhibited the highest elemental percentage, with a consistent trend of decreasing content towards the periphery.

**Table 1:** Summary of SEM-EDS results comparing elemental composition and morphological characteristics across different cell groups.

Cell group	Peripheral elemental composition compared to CD133+ (%)	Central elemental composition compared to CD133+ (%)	Morphological characteristics
CD133+	Baseline	Baseline	Large, rounded, protrusions
CD133-	Low C, high O, slightly high P	Low C, high O, low K	Smaller, more elongated
CD133+/KLF4-	Low C, high N, slightly low S	Low N, low S, low K	Flattened, decreased filapodia
CD133+/SHH-	High O, low S	Similar to CD133+	Flattened
CD133+/Hif1α-	Low S, low K	Low N, high K, low S	Flattened, smaller

Elemental variations in peripheral and central regions are presented relative to the CD133 + group. Morphological characteristics describe overall cell shape and structural features observed in each group.

### Morphometric evaluation of cell shape alterations response to KLF4, SHH, and HIF1α silencing

**Fig. 2:**
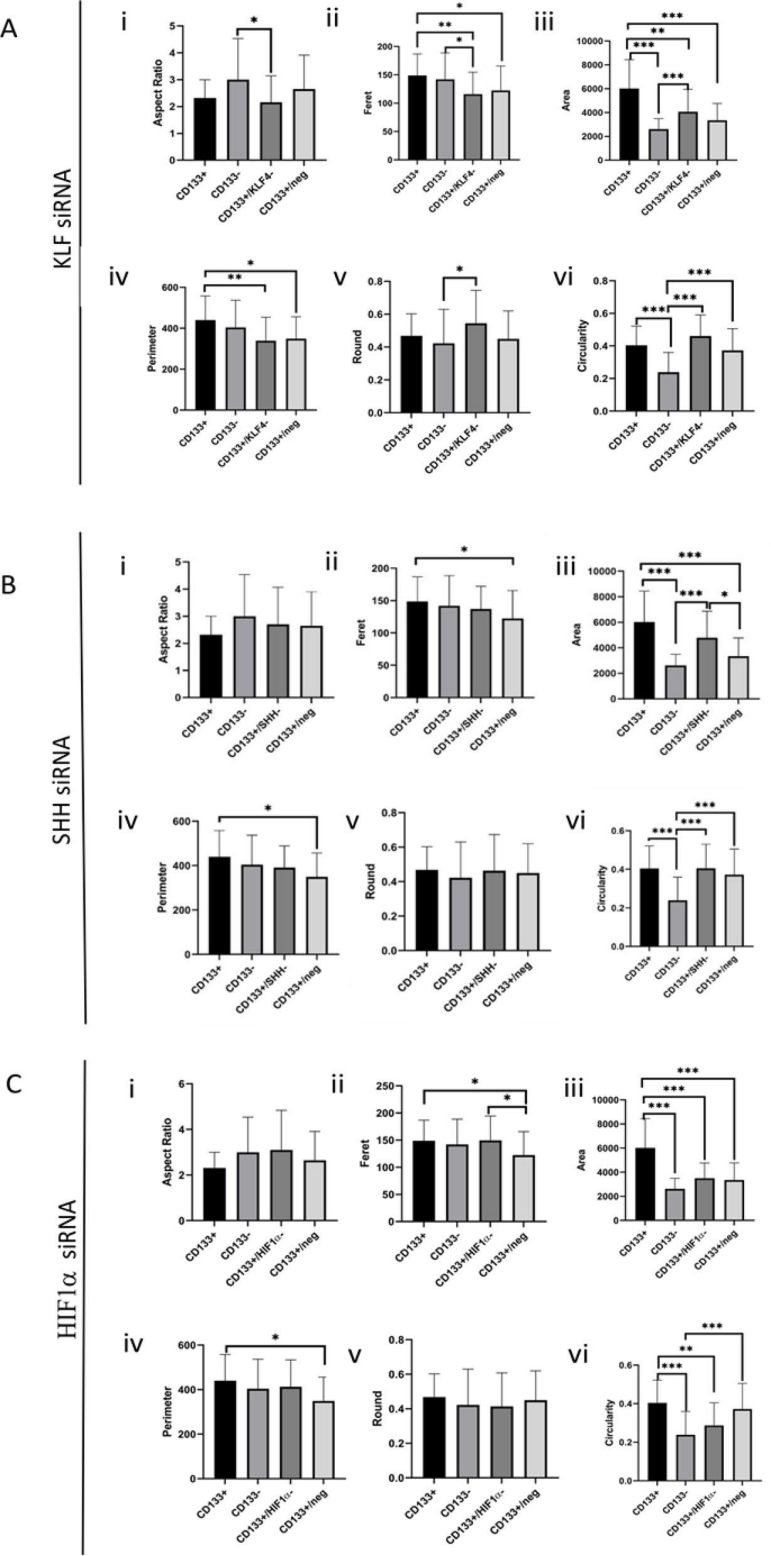
Quantitative morphometric analysis of melanoma CD133 + cells and corresponding gene-silenced derivatives. Morphological parameters were quantified for CD133+, CD133-, CD133+/neg, and CD133 + cells transfected with siRNAs targeting KLF4 (**A**), SHH (**B**), or HIF1α (**C**). Measurements included: (i) aspect ratio, (ii) Feret’s diameter, (iii) cell area, (iv) perimeter, (v) roundness, and (vi) circularity. Data are presented as mean ± standard deviation (SD), and statistical significance was set at *p* < 0.05, *p* < 0.01, *p < 0.001.

Morphological assessments of the cytoskeleton obtained through F-actin staining were further extended. F-actin represents one of the cytoskeletal elements, and evaluations of cellular attributes such as area, perimeter, and more were conducted in the study. Consequently, various parameters including aspect ratio, feret, area, perimeter, roundness, and circularity values of the cell groups were compared (Fig. 2).

Aspect ratio of CD133 + and CD133- cell groups were similar. However, the CD133+/KLF4- cell group was statistically different from the CD133- cell group and exhibited a lower aspect ratio (*p* < 0.05). In contrast, no significant difference was observed in the comparison between CD133+/SHH- and CD133+/HIF1α cell groups when compared to both CD133 + and CD133- cell groups. The highest mean values were observed in the CD133+/HIF1α- group. Following KLF4, SHH, and HIF1α siRNA treatments, the major/minor axis data displayed similar trends.

Feret values did not differ significantly between the CD133 + and CD133- cell groups. However, the CD133+/KLF4- cell group was statistically different from both the CD133 + and CD133- cell groups and exhibited lower Feret values (*p* < 0.01 and *p* < 0.05). On the other hand, the CD133+/SHH- and CD133+/HIF1α- cell groups showed similar values to both the CD133 + and CD133- cell groups.

The area of CD133 + cell group is statistically distinct from the other groups (*p* < 0.001). The highest mean area value was observed in the CD133 + cell group. In contrast, the CD133+/KLF4- cell group has a smaller area compared to the CD133 + group but a larger area compared to the CD133- group (*p* < 0.01 and *p* < 0.001). The CD133+/SHH- cell group has a larger area than the CD133- cell group (*p* < 0.001). In the CD133+/HIF1α- cell group, a decrease in area values was observed.

The highest perimeter mean value among the groups is the CD133 + cell group. However, there is a statistical difference between the CD133 + and CD133+/nsiRNA cell groups (*p* < 0.05). Additionally, the CD133+/KLF4- cell group has a higher perimeter value compared to the CD133 + cell group (*p* < 0.01).

There was no statistically significant difference between the CD133 + and CD133- cell groups in round values. However, the CD133+/KLF4- cell group was found to be higher compared to the CD133- cell group (*p* < 0.05).

There is a statistical difference between the CD133 + and CD133- cell groups, with the CD133 + cell group exhibiting higher values in circularity (*p* < 0.001). The CD133+/KLF4- and CD133+/SHH- cell groups are also different from the CD133- cell group, with the CD133+/KLF4- cell group having higher values. The CD133+/HIF1α- cell group is statistically distinct from the CD133 + cell group, and after HIF1α siRNA treatment, cellular circularity data decreased, suggesting a reduction in cell protrusions in this group.

### Biochemical alterations identified by ATR-FTIR after gene silencing

**Fig. 3:**
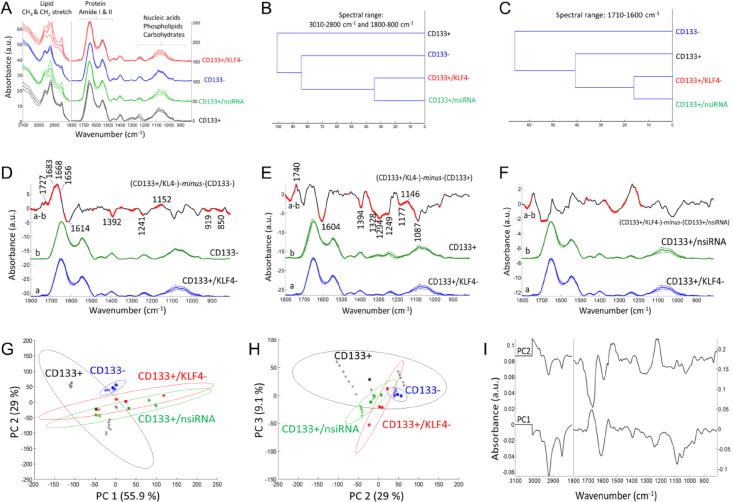
The ATR-FTIR data after the silencing of KLF4 gene. (**A**) Mean absorbance spectrum of each cell group (black color: CD133 + cells; blue color: CD133- cells; green color: the CD133 + group treated with negative siRNA; red color: the CD133 + group treated with KLF4 siRNA). (**B**, **C**) Hierarchical classification of cell groups, performed based on the Euclidean distances between the spectra. The FT-IR difference spectrum of (D) ΔA=(CD133+/KLF4-)-minus-(CD133-), (E) ΔA=(CD133+/KLF4-)-minus-(CD133+) and (F) ΔA=(CD133+/KLF4-)-minus-(CD133+/nsiRNA) and Student’s t-test computed at every wavenumber with a significance level of α = 0.1%. Red thicker lines indicate statistically significant differences between the mean spectra. (G, H) Principal component analysis (mean-centered) for PC1, PC2 and PC3 plots with the percentage of total variance in each PC. (I) The PC1 and PC2 loading spectra.

**Fig. 4:**
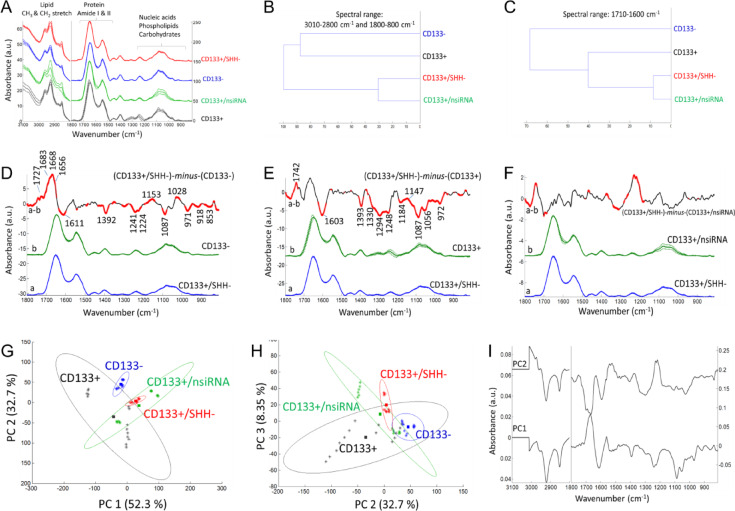
The ATR-FTIR data after the silencing of SHH gene. (**A**) Mean absorbance spectrum of each cell group (black color: CD133 + cells; blue color: CD133- cells; green color: the CD133 + group treated with negative siRNA; red color: the CD133 + group treated with SHH siRNA). (**B**, **C**) Hierarchical classification of cell groups, performed based on the Euclidean distances between the spectra. The FT-IR difference spectrum of (**D**) ΔA=(CD133+/SHH-)-*minus*-(CD133-), (E) ΔA=(CD133+/SHH-)-*minus*-(CD133+) and (F) ΔA=(CD133+/SHH-)-*minus*-(CD133+/nsiRNA) and Student’s t-test computed at every wavenumber with a significance level of α = 0.1%. Red thicker lines indicate statistically significant differences between the mean spectra. (G, H) Principal component analysis (mean-centered) for PC1, PC2 and PC3 plots with the percentage of total variance in each PC. (I) The PC1 and PC2 loading spectra.

**Fig. 5:**
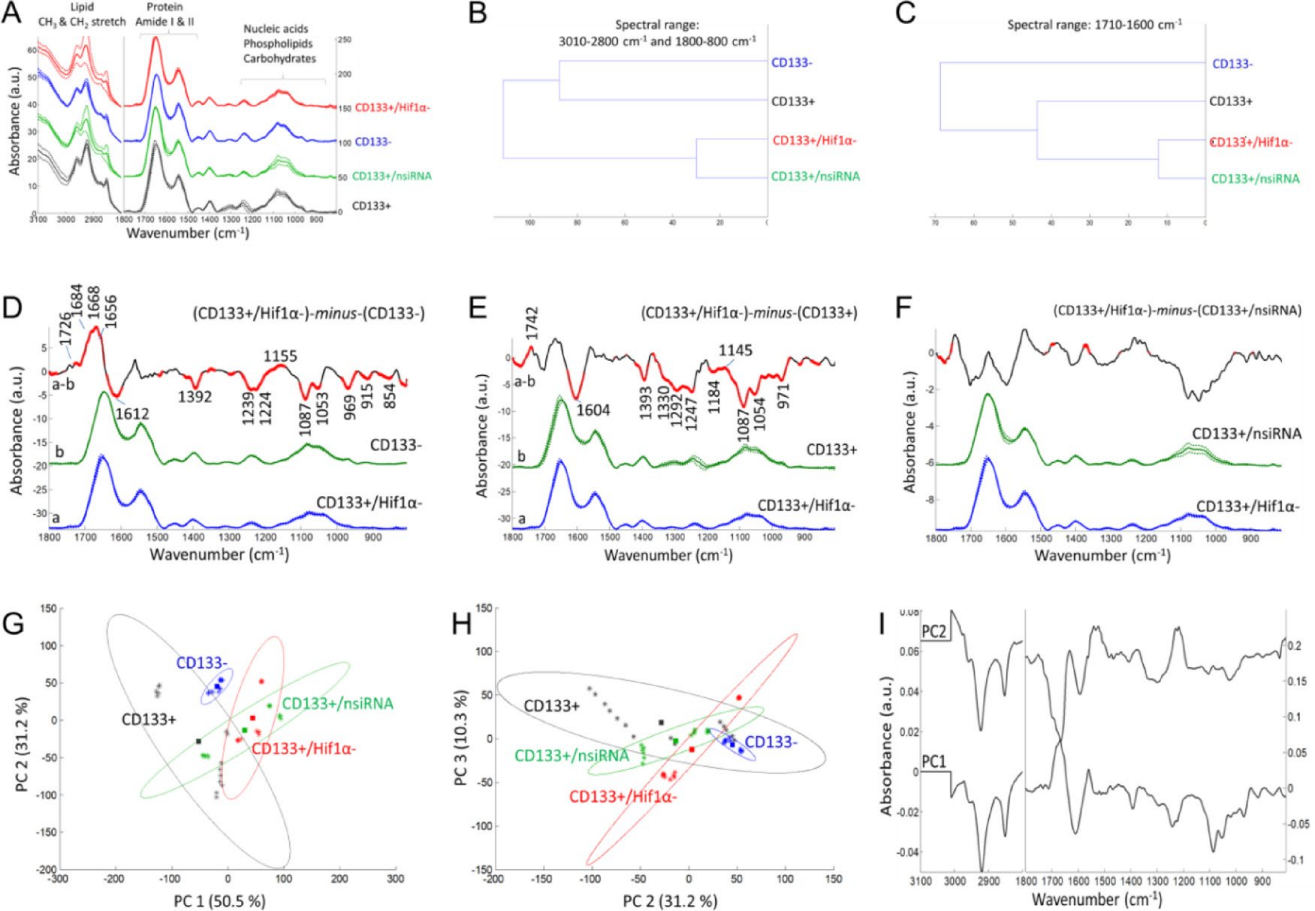
The ATR-FTIR data after the silencing of HIF1α gene. (**A**) Mean absorbance spectrum of each cell group (black color: CD133 + cells; blue color: CD133- cells; green color: the CD133 + group treated with negative siRNA; red color: the CD133 + group treated with HIF1α siRNA). (**B**, **C**) Hierarchical classification of cell groups, performed based on the Euclidean distances between the spectra. The FT-IR difference spectrum of (**D**) ΔA=(CD133+/HIF1α-)-*minus*-(CD133-), (**E**) ΔA=(CD133+/HIF1α-)-*minus*-(CD133+) and (**F**) ΔA=(CD133+/HIF1α-)-*minus*-(CD133+/nsiRNA) and Student’s t-test computed at every wavenumber with a significance level of α = 0.1%. Red thicker lines indicate statistically significant differences between the mean spectra. (**G**, **H**) Principal component analysis (mean-centered) for PC1, PC2 and PC3 plots with the percentage of total variance in each PC. (I) The PC1 and PC2 loading spectra.

The FTIR spectrum of cells exhibits signals originating from cellular components such as lipids, proteins, carbohydrates, nucleic acids etc., providing abundant information about the composition, structure, and dynamics of cellular macromolecules^28,29^. Figures 3, 4 and 5 display the spectral alterations in cell groups upon siRNA-mediated silencing of KLF4, SHH and HIF1α gene expressions, respectively. Assignments of the major IR bands for were listed in Table 2 for clarity.

The mean (average) FTIR absorbance spectra of each cellular group (Figs. 3A, 4A and 5A) represents characteristics IR signals arise mainly from functional groups of cellular macromolecules (lipids, proteins, nucleic acids, carbohydrates etc.). Accordingly, the spectral range of 3000 –2800 cm^− 1^ is contributed from the asymmetric and symmetric stretching vibrations of the CH_3_ and CH_2_ groups mainly in lipids. Besides, the bending vibrations of the CH groups give rise to weak signals (1500 –1350 cm^− 1^) and may overlap with the amino acid side chains. Absorption signals of proteins are observed in the amide I (1700 –1600 cm^− 1^), amide II (1600 –1500 cm^− 1^) and amide III (∼1350 –1250 cm^− 1^) bands. The strong amide I band is often used to obtain detailed information about protein secondary structures (α-helix, β-sheet, turn, random coils), conformation as well as total protein amount. The spectral range of 1240 –1190 cm^− 1^ is contributed by the IR peaks of phosphate groups, such as involved in phospholipids and nucleic acids (peaks at 1240 –1190 cm^− 1^ and around 1087 cm^− 1^ due to the antisymmetric and symmetric stretching vibrations of phosphate groups, respectively). The spectral range of 1160 –900 cm^− 1^ is mainly dominated by the IR peaks of carbohydrate derivatives due to the COH coupling, C-O and C-C vibrations (for band assignments see refs^29–35^.

**Table 2:** Major FTIR band positions from Figs. 3, 4 and 5 and band assignments based on the refs^29,31–35^.

FTIR band position (cm^− 1^)	Definition for band assignments
2959, 2874	*ν*_as_(CH_3_) and *ν*_s_(CH_3_), respectively: in lipids and proteins
2924, 2853	*ν*_as_(CH_2_) and *ν*_s_(CH_2_), respectively: mainly in lipids
1742, 1727	lipid esters ν(C = O): phospholipids, cholesterol esters and triglycerides
1683,1668, 1656	turn, loops, α-helix/unoredered, respectively: protein secondary structures in amide I band due to *v*(C = O)
1614 − 1580	carboxyl group *v*_as_(COO^−^), δ(NH_2_): such as Asp/Glu, Asn/Gln
1550 − 1530	protein amide II band (N–H bending, C–N stretching)
1467, 1452	CH_2_ and CH_3_ scissoring and bending: mainly lipids with small contribution from proteins
1392	carboxyl group *v*_s_(COO^−^): free amino acids, fatty acids
1350 − 1250	protein amide III band (coupled C-N stretching and NH bending vibrations)
1240 − 1190	*v*_*a*s_(PO_2_^−^): phospholipids, nucleic acids
1177	C-OH groups of serine, threonine and tyrosine in proteins, triglyceride, cholesterol
1153, 1028	*ν*(C-O) of glycogen, phosphate-sugar backbone of nucleic acids
1087	*v*_s_(PO_2_^−^): phospholipids, nucleic acids
1056, 1028	ν_s_(C-O) and δ(C-O): C-OH groups of polysaccharides, glycogen
972	*v*(PO_4_^−^) of nucleic acids; C-O deoxyribose, C-C stretching of DNA backbone
916	ribose ring vibration

ν, stretching; δ, bending; as, antisymmetric; s, symmetric.

The FTIR mean spectra (Figs. 3A, 4A and 5A) were used for hierarchical clustering analysis (HCA). In HCA, the groups are compared in Figs. 3B and 4B, and 5B, where the similarities between cell characteristics (proteins, lipids, carbohydrates, nucleic acids etc.) are indicated in the whole spectrum. According to the HCA results, the group treated with negative siRNA and the groups treated with target siRNA were found to be similar, while the group treated with KLF4 siRNA was more similar to the CD133- cell group (Fig. 3B). After the silencing of the SHH genes (Fig. 4B) and HIF1α genes (Fig. 5B), the CD133 + and CD133- cell groups were found to be similar and distinct from the gene silencing groups. In the comparison of cell groups, particular emphasis was also placed on the protein-based comparisons. The spectral range of 1700 –1600 cm^− 1^ (amide I) was also examined for protein-specific analysis in HCA. Accordingly, the group treated with negative siRNA and the groups treated with target siRNA were found to be similar, while the CD133 + cell group was more similar to the siRNA-treated groups (Figs. 3C and 4C, and 5C).

In order to determine the small absorbance changes in the IR spectrum, the differences between groups are illustrated in the FTIR difference spectra with Student’s t-test obtained from the subtraction of means (KLF4, Fig. 3D, E, F; SHH, Fig. 4D, E, F; HIF1α, Fig. 5D, E, F). To obtain the FTIR difference spectrum ΔA=(condition1)-*minus*-(condiiton2), the mean absorbance spectrum of CD133- cells, CD133 + cells and the group treated with negative siRNA (denoted as CD133+/nsiRNA) was separately subtracted from the mean absorbance spectrum of siRNA treated CD133 + cells. For instance, the mean absorbance spectrum of CD133- cells was subtracted from the mean absorbance spectrum of CD133 + group treated with KLF4 siRNA (denoted as CD133+/KLF4-), and the resultant difference spectrum is so-called (CD133+/KLF4-)-*minus*-(CD133-) (Fig. 3D); in that difference spectrum, the positive IR peaks correspond to the increased part of the cellular components of the group treated with KLF4 siRNA while negative peaks reflect its reduced cellular components. Accordingly, when compared to the CD133- cells, the KLF4 silencing CD133 + group (Fig. 3D) exhibits the high protein content (positive IR signals absorbed at 1683, 1668 and 1656 cm^− 1^ consistent with protein-related amide I vibrations) and lipid esters (positive peak at 1727 cm^− 1^ due to C = O vibrations) while it has less amount of negatively charged amino acids (broad negative peaks at 1614 and 1392 cm^− 1^ due to carboxyl group COO^−^ vibrations). Similarly, the SHH silencing (Fig. 4D) and HIF1α silencing (Fig. 5D) CD133 + groups also exhibit the high protein content and lipid esters but they have the low amount of negatively charged COO^−^ groups and nucleic acids (negative peaks at 1241, 1224, 1087 and 971 cm^− 1^ due to the vibrations of phosphate groups). The content of the carbohydrate groups (~ 1153, 1053, 1028 cm^− 1^) are also altered in both SHH silencing (Fig. 4D) and HIF1α silencing (Fig. 5D) CD133 + groups. Interestingly, when compared to the CD133 + cells, each spectral profile of target genes silencing CD133 + cells (Figs. 3E, 4E and 5E) displays the broad negative amide III band (1350 –1250 cm^− 1^) due to coupling of C-N stretching and NH bending vibrations in proteins in the form of fibre/filament^30,36^. This most likely suggests the low amount of some filamentous protein structures, such as F-actin protein (representing the cell cytoskeleton) upon silencing of the target genes in CD133 + cells. This data is harmonious with the F-actin staining results in Fig. 2. Target genes silencing CD133 + groups also have the high content of lipid esters (positive peak around 1742 cm^− 1^), the low amount of negatively charged COO^−^ groups (broad negative peaks around 1603 and 1394 cm^− 1^), and low nucleic acids content (negative peaks at 1248, 1087 and 972 cm^− 1^). Furthermore, the amount of the carbohydrate derivatives (multiple positive and negative peaks at 1150 –1000 cm^− 1^) is largely altered in both SHH silencing (Fig. 4E) and HIF1α silencing CD133 + groups (Fig. 5E) (but not significant in KLF4 silencing group, Fig. 3E). However, when compared to the group treated with negative siRNA, both KLF4 silencing (Fig. 3F) and SHH silencing (Fig. 4F) CD133 + cells have the high amount of nucleic acids (broad positive peak at 1250 –1190 cm^− 1^) (not significant in HIF1α silencing group, Fig. 5F).

Mean centred principal component analysis (PCA) was conducted to assess the similarities and differences among the cell groups (Fig. 3G, H for KLF4; Fig. 4G, H for SHH; Fig. 5G, H for HIF1α). According to the PCA results, the siRNA-treated groups are well separated from the CD133 + cell group, all with positive PC1 scores (55.9% for KLF4; 52.3% for SHH; 50.5% for HIF1α). Their PC1 loading spectra generally exhibit the negative peaks at 3000 –2800 cm^− 1^ (lipids), the positive peak at 1700 –1650 cm^− 1^ in the protein amide I band, and have multiple positive and negative peaks in the 1250 –1000 cm^− 1^ range (nucleic acids and carbohydrate derivatives). This indicates that the siRNA-treated cells are discriminated from other groups based on variations in cellular constituents, including lipids, proteins (amide I band region), nucleic acids and carbohydrate derivatives. Based on PC2 plot, the siRNA-treated groups and CD133- groups are separated both from the CD133 + group and CD133+/nsiRNA group with positive PC2 scores (29% for KLF4; 32.7% for SHH; 31.2% for HIF1α). Their PC2 loading spectra generally exhibit the broad positive peaks at 1560 –1490 cm^− 1^, 1250 –1190 cm^− 1^, 1086 cm^− 1^, 966 cm^− 1^ (due to nitrogenous bases and phosphate groups of nucleic acids) and represent the negative peaks at 3000 –2800 cm^− 1^ (lipids) and at 1700 –1650 cm^− 1^ in the protein amide I band. This strongly indicates that the siRNA-treated groups and CD133- groups are discriminated from other groups based on variations in nucleic acids, lipids and proteins. The PCA results are in line with the spectral profile of FTIR difference spectra and HCA analysis for each group. However, the groups are discriminated to the lesser extent in the PC3 plots (9.1% for KLF4; 8.35% for SHH; 10.3% for HIF1α) (For band assignments see Refs^29,31–35^.

### X-Ray photoelectron spectroscopy reveals surface chemical alterations after gene Silencing

XPS is a semi-quantitative method used to obtain the percentage data of selected elements in a region with a surface penetration capability of approximately 10 nm. This method provided approximate data from at least three different samples for the region defined as the cell membrane and the immediately underlying region of the membrane. For each cell group, the first XPS measurement was a survey scan with a binding energy range of 1350 to -10 eV (the spectra are shown in the SI Figs. 2 and 3). For these measurements, the analyzer pass energy was set to 200 eV. In the survey spectra, oxygen, nitrogen, carbon and sulfur were the prominent elements for every cell group. In addition to these, a small amount of phosphorus was observed for the CD133 + cell group, and a small amount of silicon was observed for the CD133+/HIF1α, CD133+/SHH and CD133+/nsiRNA cell groups. High resolution measurements were performed for O 1s, N 1s, C 1s and S 2p energy levels for which the analyzer pass energy was set to 50 eV.

**Fig. 6:**
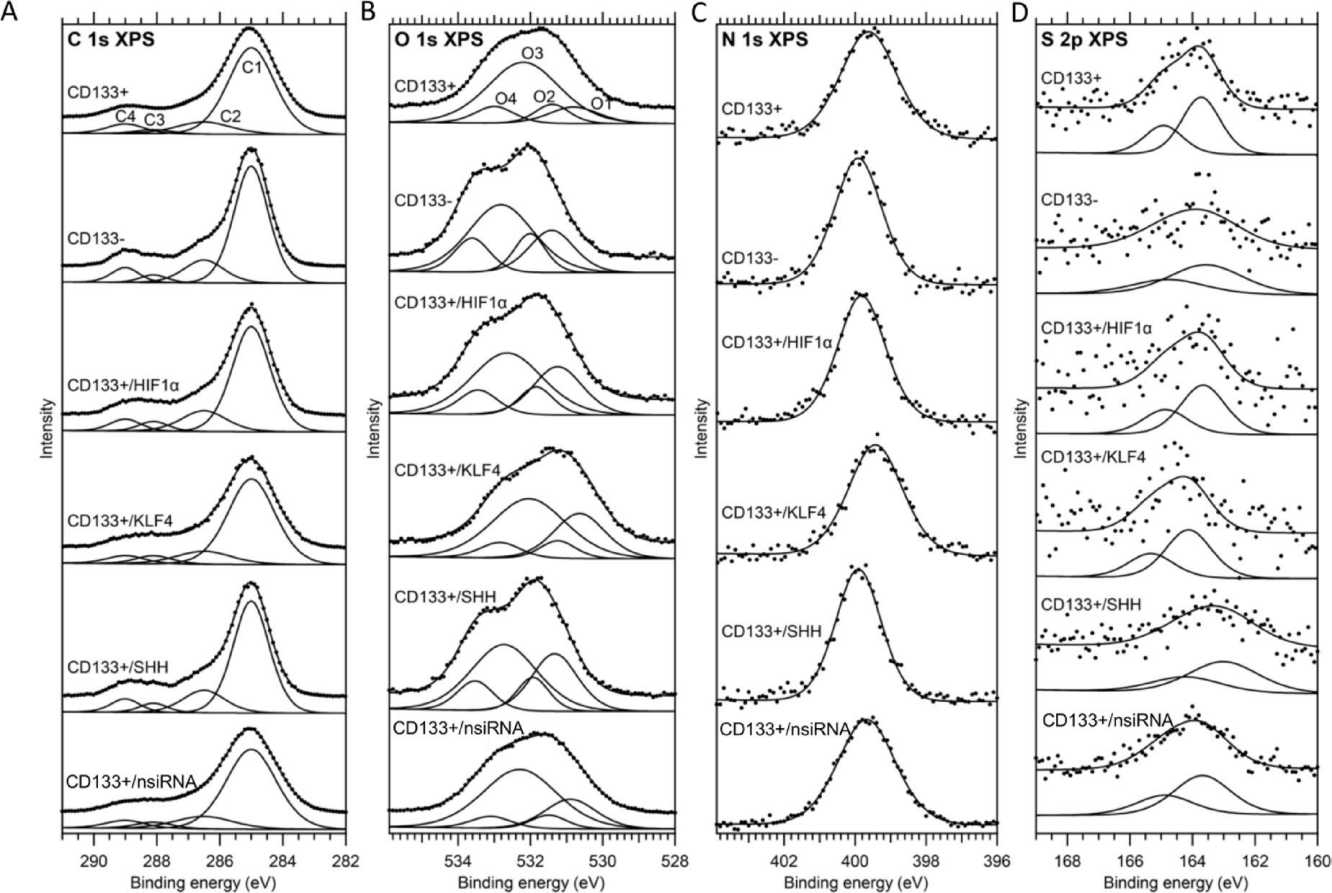
(**A**) The C 1s X-ray photoelectron spectra and the results of peak fitting for all cell groups. The spectra are normalized to the area of the highest-intensity peak (C1). (**B**) The O 1s x-ray photoelectron spectra and the results of peak fitting for all cell groups. The spectra are normalized to the area of the highest-intensity peak (O3). For each spectrum, the measurements (dots) and the resulting fit (solid line) are shifted upward to improve visibility. (**C**). The N 1s and (**D**) S 2p x-ray photoelectron spectra and the results of peak fitting for all cell groups. The spectra are normalized to the area of the highest-intensity peak. For each spectrum, the measurements (dots) and the resulting fit (solid line) are shifted upward to improve visibility.

The fitting results for the C 1s spectra are shown in the Fig. 6A. In line with the literature^37–47^the C 1s spectra were fitted with four components. The C1 component is assigned to aliphatic hydrocarbons (C_x_H_y_). The C2 component is assigned to carbon that forms a single bond with either oxygen (C-O) in alcohol and ester functional groups, nitrogen (C-N) in amide and amine functional groups or sulfur (C-S). The C3 component is assigned to the carbon in the carbonyl bond (C = O) in the amide functional group, the carbon in the carboxylate functional group, and the carbon that forms two single bonds with two oxygen atoms (O-C-O) in the acetal functional group. The C4 component is assigned to the carbon in the carbonyl bond in carboxyl and ester functional groups.

The signature of these four components was most distinctly observed in the C 1s spectrum of the CD133- cell group. Therefore, the C 1s spectrum of the CD133- cell group was fitted first and used as a reference for the other cell groups. In the fitting procedure for the CD133- cell group, the FWHM values for all components and the binding energy value for the C1 component were set free and the binding energy values for the C2, C3 and C4 components were fixed at 1.5, 3.1 and 4 eV higher binding energies compared with the C1 component^45^. Although the contributions of instrumental and lifetime broadening to the FWHM values must be equal for all C 1s components, the FWHM values for the C2, C3, and C4 components were fixed to the FWHM value of the C1 component, scaled by a constant factor, to account for inhomogeneity in the structure and possible charging effects across different cell groups. Therefore, in the fitting procedure for the other cell groups, the only free parameters were the binding energy and FWHM values for the C1 component.

The fitting results for the O 1s spectra are shown in the Fig. 6B. Four components were also used for fitting O 1s spectra^45^. The O1 component is assigned to the oxygen in the carbonyl bond of amide and equivalent oxygen atoms in the carboxylate functional groups. The O2 component is assigned to the oxygen in the carbonyl bond of ester and carboxyl functional groups. The O3 component is assigned to the oxygen that forms a single bond with the C atom (C-O) in alcohol and acetal functional groups. The O4 component is assigned to the oxygen that forms a single bond with the carbon in carboxyl and ester functional groups.

O 1s spectrum of the CD133- cell group was chosen as a reference for fitting the O 1s spectra to be consistent with the procedure used for C 1s spectra. Similar to the fitting procedure used for the C1 spectrum of the CD133- cell group, the binding energy for the O1 component and FWHM values for all components were set free while the binding energy values for the O2, O3 and O4 components were fixed at 0.6, 1.4 and 2.2 eV higher binding energies compared with the O1 component^45^. In addition, because O2 and O4 components result from different oxygen atoms of the same functional groups (ester and carboxyl), their areas were set equal. Accordingly, in the fitting procedure for the other cell groups, the only free parameters were the binding energy and FWHM values for the O1 component. Similar to the case for fitting C 1s spectra the FWHM values for the O2, O3, and O4 components were fixed to the FWHM value of the O1 component up to a constant factor.

The fitting results for the N 1s and S 2p spectra are shown in the Fig. 6C and D. In the N 1s region, only one peak is observed and this peak is assigned to nitrogen in amide or amine functional groups^38,39^^41^–^43,45,47,48^. In the S 2p region, only one doublet is observed and this doublet is assigned to sulfur in thiol (-SH) or disulfide (-S-S-) species^49–54^. For the S 2p spectra, the binding energy difference (1.2 eV) and area ratio (1:2) for the 1/2 and 3/2 components were fixed to common literature values^55^. In the Table 3 fitting results for the C 1s and in the Table 4 fitting results for O 1s, N 1s and S 2p spectra are presented.

**Table 3:** The binding energy, FWHM, and total carbon-normalized peak area for different carbon species for all cell groups.

Cell Groups	C1 (1s)	C2 (1s)	C3 (1s)	C4 (1s)
CD133+	285.00 1.74 77.7%	C1 + 1.50 C1*1.16 12.6%	C1 + 3.10 C1*0.76 2.7%	C1 + 4.00 C1*0.79 6.9%
CD133-	285.00 1.29 72.7%	C1 + 1.50 1.50 16.4%	C1 + 3.10 0.98 3.6%	C1 + 4.00 1.02 7.3%
CD133+/HIF1α-	285.00 1.43 72.0%	C1 + 1.50 C1*1.16 16.5%	C1 + 3.10 C1*0.76 5.0%	C1 + 4.00 C1*0.79 6.5%
CD133+/KLF4-	285.00 1.76 75.6%	C1 + 1.50 C1*1.16 13.4%	C1 + 3.10 C1*0.76 5.4%	C1 + 4.00 C1*0.79 5.6%
CD133+/SHH-	285.00 1.35 71.8%	C1 + 1.50 C1*1.16 16.8%	C1 + 3.10 C1*0.76 4.5%	C1 + 4.00 C1*0.79 6.8%
CD133+/nsiRNA	285.00 1.89 75.8%	C1 + 1.50 C1*1.16 13.7%	C1 + 3.10 C1*0.76 4.5%	C1 + 4.00 C1*0.79 6.0%

The value at the top is the binding energy, the value in the middle is the FWHM (both in eV), and the value at the bottom is the peak area that is normalized to the total amount of carbon.

**Table 4:** The binding energy, FWHM, and total carbon-normalized peak area for different oxygen species and nitrogen and sulfur for all cell groups.

Cell Groups	O1 (1s)	O2 (1s)	O3 (1s)	O4 (1s)	*N* (1s)	S (2p_3/2_)
CD133+	530.79 1.62 6.3%	O1 + 0.60 O1*0.69 5.0%	O1 + 1.40 O1*1.45 33.5%	O1 + 2.20 O1*0.79 O2	399.62 1.87 4.9%	163.72 1.45 0.3%
CD133-	531.41 1.46 11.0%	532.01 1.01 7.0%	532.81 2.12 25.3%	533.61 1.15 O2	399.91 1.58 8.4%	163.57 2.79 0.4%
CD133+/HIF1α-	531.24 1.6 14.5%	O1 + 0.60 O1*0.69 5.8%	O1 + 1.40 O1*1.45 26.8%	O1 + 2.20 O1*0.79 O2	399.82 1.58 11.4%	163.66 1.68 0.3%
CD133+/KLF4-	530.64 1.66 15.4%	O1 + 0.60 O1*0.69 4.2%	O1 + 1.40 O1*1.45 29.4%	O1 + 2.20 O1*0.79 O2	399.43 1.80 11.4%	164.12 1.72 0.3%
CD133+/SHH-	531.33 1.49 15.0%	O1 + 0.60 O1*0.69 6.0%	O1 + 1.40 O1*1.45 24.9%	O1 + 2.20 O1*0.79 O2	399.91 1.51 10.4%	163.01 2.64 0.5%
CD133+/nsiRNA	530.89 1.67 11.7%	O1 + 0.60 O1*0.69 3.7%	O1 + 1.40 O1*1.45 33.8%	O1 + 2.20 O1*0.79 O2	399.67 1.90 9.8%	163.69 2.13 0.4%

The value at the top is the binding energy, the value in the middle is the FWHM (both in eV), and the value at the bottom is the peak area that is normalized to the total amount of carbon.

## Discussion

In the present study, the silencing of HIF1α, SHH, and KLF4 in melanoma CSCs resulted in marked alterations in cell morphology, cytoskeletal organization, and molecular profile. Specifically, the decrease in PFN1 expression following HIF1α silencing was accompanied by reduced F-actin organization, suggesting a disruption of cytoskeletal integrity. These changes were further supported by FTIR spectroscopy, which revealed a decline in amide III band intensity, and by morphometric analysis, which showed decreased circularity and area in gene-silenced cells.

Profilin 1 (PFN1) is a key regulator of actin polymerization that facilitates the conversion of G-actin to F-actin and orchestrates cytoskeletal organization. It also plays a role in signal transduction, cell migration, and intracellular trafficking. Reduced PFN1 expression in HIF1α-silenced cells, along with decreased F-actin staining intensity, suggests impaired actin filament formation. This finding is consistent with studies showing that PFN1 downregulation disrupts actin dynamics in glioblastoma and pancreatic cancer cells^56,57^.

Morphological assessment revealed significant differences between CSCs and non-CSCs, as well as among gene-silenced groups. Following siRNA application, cell area and circularity were significantly reduced, indicating cytoskeletal reorganization. The aspect ratio, which reflects the degree of cellular polarity and potential for mitotic activity, increased in gene-silenced groups, particularly in CD133 + cells. This suggests a shift away from the rounded morphology typical of stem-like cells, supporting a loss of stemness characteristics upon gene silencing.

FTIR spectroscopy has made a significant impact due to its versatility in detecting biochemical and biological characteristics in biological samples (cells, tissues, body fluids etc.). It can be employed to distinguish between healthy and pathological conditions by identifying comparative differences in the acquired data. Furthermore, characterization studies conducted on different CSCs have also demonstrated the potential utility of FTIR spectroscopy in cancer research^58,59^. In the current study, FTIR analysis were performed on malignant melanoma CSCs and non-CSCs. Additionally, specific genes were silenced, and spectral differences were identified. Based on the FTIR spectral profile, the cell groups subjected to siRNA treatment showed pronounced differences compared to both CD133 + and CD133- cell groups but exhibited similarity to the CD133+/nsiRNA cell group. Gene-silenced CD133 + cells exhibited reduced intensity at the amide III band, which may reflect changes in the overall protein secondary structure and cytoskeletal remodeling, including decreased F-actin organization, as supported by immunofluorescence analysis (Fig. 1). This suggests that the biochemical profile captured by FTIR reflects underlying cytoskeletal alterations. Additionaly, target genes silencing CD133 + cells have the high content of lipid esters (positive IR peak around 1742 cm^[- [1^), but have the low amount of negatively charged COO^-^ groups (negative peaks at 1603 − 1580 and 1394 cm^[- [1^), and low amount of nucleic acids. This increase in lipid ester signals may indicate enhanced membrane remodeling or metabolic reprogramming, processes often associated with CSC differentiation and reduced stemness. The differences in gene expression observed in silenced cells may result from the suppression of key signaling pathways. While some overlap exists with the comparison groups, these cells likely exhibit distinct characteristics due to both siRNA delivery and its impact on signaling pathways.

SEM imaging confirmed distinct morphological alterations in gene-silenced CSCs. In addition to surface visualization^60–62^SEM-EDS enabled semi-quantitative elemental profiling, revealing differential distributions of carbon, nitrogen, oxygen, sodium, and potassium among groups^13–15,63–65^. The electron beam penetration depth in SEM/EDS is reported to be in the range of 10–100 nm for a wide variety of nanomaterials with energies above 2 kV. Due to the penetration depth, the elemental ratios may differ between central and peripheral regions. While SEM-EDS has primarily been applied in microbiological and mineralized tissue studies^66–69^recent work has explored its utility in distinguishing cancer from non-cancer cell types^14^. In another study, glioblastoma and astrocyte cell lines were compared, and the differences between cancer and non-cancer cells were investigated using this method^15^. In this study, CSCs and non-cancer stem cancer cells were used as the control group and were compared with siRNA applications. The sampling depth of this method is approximately 10 nm^70^. Variations in the electronegativity of functional groups, such as CH_x_, C-O, C = O, and O-C = O, based on carbon atoms can be demonstrated through photoelectron energy shifts^27,70^. In this study, our experimental groups were compared using the same methodology in a semi-quantitative manner. The non-CSC group showed higher nitrogen levels, while siRNA application resulted in a decrease in carbon content.

XPS further supported these observations, showing increased amide-related signals (C3 and O1) in silenced cells. The differences in C2 and N levels between CD133 + and CD133- cells suggest underlying shifts in membrane composition and protein content. Consistent with the SEM-EDS results, XPS analysis revealed prominent levels of carbon, oxygen, nitrogen, and sulfur. Additionally, consistent with the literature, the N 1s peaks are prominent in the XPS measurements of the matrigel due to the presence of two main components (SI Fig. 4), laminin and collagen^71^. Hydrocarbon content decreased following SHH and HIF1α silencing, while C2 and N levels showed significant differences between the CD133 + and CD133- groups, with higher concentrations in the CD133- group. An increase in amide-related signals (C3 and O1) was observed in XPS after gene silencing. While this aligns with the reduced amide III intensity in FTIR, differences in sampling depth and surface sensitivity between techniques should be considered. It should be noted that both XPS and SEM-EDS have intrinsic limitations in spatial resolution and sampling depth. XPS, with a typical sampling depth of approximately 10 nm, is sensitive to surface and near-surface chemical states, while SEM-EDS, operating at the micron scale, provides bulk-average elemental information from the interaction volume rather than discrete subcellular compartments. Therefore, our interpretations are restricted to overall surface or near-surface compositional trends, and do not imply precise intracellular localization of elements.

The integration of molecular, spectroscopic, and imaging data provides a comprehensive perspective on how silencing of KLF4, SHH, and HIF1α affects melanoma CSCs. Morphological changes were supported by alterations in F-actin organization, FTIR-based biochemical shifts, and variations in elemental surface composition detected via SEM-EDS and XPS. The concordance among these distinct analytical approaches reinforces the reliability of our findings and highlights the interplay between transcriptional regulation and CSC plasticity. Overall, these results emphasize the importance of targeting cytoskeletal and metabolic pathways as part of combinatorial strategies to disrupt CSC maintenance and enhance therapeutic efficacy.

This study demonstrates that silencing KLF4, SHH, and HIF1α in melanoma CSCs leads to coordinated changes in cytoskeletal structure, molecular composition, and cell morphology, reflecting a loss of stem-like properties. Spectral and elemental alterations, particularly in amide and lipid content, highlight the molecular consequences of targeting these pathways. These findings contribute to the growing understanding of CSC biology and suggest that combining cytoskeletal and metabolic interventions may offer promising avenues for more effective cancer therapies.

## Materials and methods

### Cell culture

CHL-1 human melanoma cell lines (ATCC^®^ CRL-9446TM) were kindly provided by Prof. Dr. Cigir Biray Avci (Ege University, Izmir). These cells were cultured at 37 °C in a 5% CO_2_ environment in Eagle’s Minimum Essential Medium (EMEM, Biowest L0416) supplemented with 10% fetal bovine serum (Biowest, S1810). Passage 6–8 was utilized for flow cytometry sorting purposes.

### Fluorescence-activated cell sorting (FACS)

To detach cells adhered to the flask surface, trypsinization was employed, followed by dilution to a concentration of 10^6^ cells/ml. Subsequently, cells were subjected to incubation with a phycoerythrin (PE)-labeled CD133 antibody (Miltenyi Biotec Ltd.) and DAPI for 15 min at + 4 °C, followed by washing with PBS containing 1% dialyzed FBS. As a control, cells not exposed to the antibody were included. Cells positive for CD133 were categorized as CSCs, while CD133-negative cells were designated as NCSCs. Cells positive for CD133 (referred to as CD13+; cancer stem cell-enriched fraction) were categorized as CSCs, while CD133-negative cells (referred to as CD133-; non-cancer stem cell fraction) were designated as NCSCs. Cell enumeration was conducted using the Muse^®^ Cell Analyzer.

### Matrigel coating

Matrigel was applied to the surface prior to cell seeding. This was achieved by pre-chilling the required materials and utilizing them, ensuring the even dispersion of the liquid Matrigel across the surface during the coating process. Samples were incubated on ice with gentle shaking to ensure uniform distribution. Polymerization was initiated by incubating the samples at 37 °C for 20-minute duration proving adequate for complete polymerization.

### Small interfering RNA (siRNA) transfection

To downregulate the expression of HIF1α, KLF4, and SHH genes, siRNA treatment was administered to CD133 + malignant melanoma CSCs. The siRNA concentration of 25 nM for SHH was selected based on prior dose-response optimizations reported in our previous work^7,72^where this dose provided the most effective gene knockdown with minimal off-target effects. In contrast, 5 nM concentrations were adequate for KLF4 and HIF1α silencing. As a procedural standard, a negative control siRNA at 25 nM was included in the experiments. A 25 nM negative control siRNA was used across all groups to ensure uniformity in transfection conditions. Previous optimization studies demonstrated no phenotypic or molecular differences between 5 nM and 25 nM negative controls in the same melanoma CSC model (Ozdil et al., 2025), supporting the use of this concentration as a neutral baseline. Cellular evaluations were conducted 24 h following siRNA treatment.

### RT-PCR

Total RNA was extracted from cell groups using a Roche isolation kit following manufacturer instructions. RNA concentration and purity were measured spectrophotometrically. Gene expression was quantified via RT-PCR using a Roche custom panel, with ACTB as the reference gene and PFN1/PFN2 as targets. Expression levels were analyzed using the 2^ΔΔCt^ method, and results were considered significant at ≥ 2-fold change. Experiments were performed in triplicate. The hierarchical heatmap visualization was generated using Clustvis^73^.

### F-actin staining

F-actin organization was evaluated via phalloidin staining 24 h after siRNA transfection. Cells were fixed with 4% paraformaldehyde, permeabilized with 0.25% Triton X-100, and stained with Phalloidin 488 (ab176753). Nuclei were counterstained with DAPI, and samples were mounted with Fluoroshield (ab104139). Images were captured using an Olympus BX-51 fluorescence microscope.

### SEM/EDS

Before SEM imaging and analysis, the fixed samples were thoroughly dried in ambient air. To render the cell surfaces conductive for SEM imaging, all samples were coated with a 6 nm layer of gold-palladium and vacuum-coated using a Leica EM ACE600 (Leica Microsystems, Germany) sputter coater. This process took approximately 30 min and was performed in an argon gas environment. Imaging resolution and acceleration voltage were set at 0.9 nm and 1 kV, respectively. SEM/EDS imaging was conducted using a Thermo Scientific Apreo S LoVac SEM (ThermoFisher Scientific, U.S.A.) equipped with a Schottky field emission gun. High vacuum mode was selected for image acquisition. Measurements were carried out with an acceleration potential of 30 kV and a maximum beam current of 50 nA. For elemental spectrum analysis from each cell, an EDS detector (AMETEK, USA) from EDAX was utilized. An accelerating voltage of 5 kV was applied for all electron beam interactions with the cells. The micron-scale interaction volume of EDS results in spectra that reflect the overall elemental composition of the sampled area, limiting the resolution of the fine-scale distribution of elements inside intracellular compartments.

### ATR-FTIR spectroscopy

#### FTIR data acquisition

The cell groups were washed three times with 0.9% NaCl solution prior to IR spectroscopy measurements. Then, measurements were performed by using the FTIR spectrometer (PerkinElmer UATR Two, UK) equipped with an ATR unit (1 reflection with a diamond crystal) and a DLATGS detector. For each measurement, 2 µl were taken from a solution containing 2 × 10^5^ cells/ml and left to dry for approximately ten minutes. The drying process was monitored by spectra to ensure the removal of water O-H signals. At the end of the drying process, a minimum of four spectra were recorded in the mid-infrared range of 4000 –800 cm^− 1^ at room temperature. The experiment was conducted in triplicate. All FTIR measurements were performed using a commercial PerkinElmer Spectrum Two UATR instrument with the following parameters: 64 scans, 4 cm⁻¹ resolution, and a spectral range of 4000–800 cm⁻¹. Under these settings, each complete measurement took approximately 6 min, which includes the total time required for all 64 scans. The time per individual scan is therefore well below 1 s. Moreover, there was an approximately 6-minute interval between successive measurements, allowing the sample sufficient time to return to ambient temperature. The PerkinElmer UATR source uses low-intensity IR radiation and does not induce significant local heating under these conditions. Throughout the experiment, no visible changes were observed in the sample appearance or spectral features indicative of thermal denaturation. The diamond ATR unit did not apply active heating, and no increase in local sample temperature was observed. Samples were dried and fully equilibrated prior to acquisition to ensure thermal and spectral stability. The spectral background was recorded as air spectrum when the ATR unit is clean. Cells were air-dried at room temperature for approximately 10 min prior to measurement. Drying progress was monitored spectrally by tracking the attenuation of water O–H absorbance (~ 3400 cm⁻¹) to ensure consistency. We avoided heat or vacuum drying to minimize structural distortion. This approach is based on our prior work^59,74^which confirmed that air-drying under these conditions preserves the native spectral profile of biological samples.

### FTIR data analysis

The spectral pre-processing, mean spectrum, principal component analysis (PCA), hierarchical classification analysis (HCA), difference spectrum and Student’s t-test were employed by using the software *Kinetics* running under MATLAB (R2011b), as described in our previous studies^28,29^. Each spectrum was first normalized by integrating the area under the curve between 1710 and 1485 cm⁻¹, covering the amide I and II bands, to account for thickness and concentration variability. Baseline correction was applied using piecewise linear interpolation across 13 anchor points chosen from non-absorbing spectral regions (3970, 3700, 2800, 2700, 1800, 1758, 1485, 1360, 1274, 1190, 945, 895, and 800 cm⁻¹). No smoothing (such as Savitzky-Golay filtering or polynomial fitting) was applied, to avoid artificially altering spectral features. Afterwards, baseline corrected spectra were normalized for equal area between 1710 and 1485 cm^− 1^, as performed in our previous studies^22,29,74^. This approach, previously validated in our FTIR studies on cancer cells to improve comparability across spectra. Although more automated methods such as rubber band or polynomial fitting exist, the current approach enabled us to minimize biological noise and maintain consistency across all cell groups. Mean centered PCA was employed for the combined regions of 3010 –2800 cm^− 1^ and 1800 − 800 cm^− 1^ using fully pre-processed (baseline-corrected and normalized) all absorbance spectra. HCA was performed using Ward’s clustering algorithm for the 1800 –800 cm^− 1^ range and for the combined regions of 3010 –2800 cm^− 1^ and 1800 − 800 cm^− 1^ using fully pre-processed (baseline-corrected and normalized) mean absorbance spectra. In HCA, the distances between the spectra were computed as Euclidean distance. For calculation of mean (average) absorbance spectrum, FTIR data set of minimum 12 spectra recorded for each cell group (siRNA treated groups and control groups) were averaged after fully pre-processing (baseline corrected and normalized), and this average spectrum was called ‘mean’. Later on, the mean absorbance spectrum of each cell group was subtracted from the mean absorbance spectrum of siRNA treated cells, such that FTIR difference spectrum corresponds to ΔA=(condition1)-*minus*-(condition2). Student’s t-test was also computed at every wavenumber using the 12 individual spectra, which allows a statistical comparison. Each experimental group consisted of three independent biological replicates, derived from separate cell cultures. From each replicate, at least four spectra were acquired, resulting in a minimum of 12 spectra per group for statistical analysis. In the difference spectrum, the red points refer to wavenumbers where the difference is significant for α = 0.1%.

### X-ray photoelectron spectroscopy (XPS)

Prior to applying the copper tape, carbon tape was placed on the coverslip, and then copper tape was affixed on top. This arrangement was coated with Matrigel, and cells were cultured on it. After fixation and PBS washing steps, the samples were rinsed three times with distilled water. Subsequently, a drying process was carried out, and the samples were mounted onto a plate. XPS data were acquired from three independent biological replicates, each prepared and fixed separately to ensure biological reproducibility. Vacuum procedures were performed within the device.

For these experiments, a K-Alpha X-Ray photoelectron spectrometer (Thermo Fisher Scientific, UK) was employed. Measurements were conducted using Al K monochromatic (1486.68 eV) X-ray source, utilizing a 128-channel detector and a 180° hemispherical analyzer. The X-ray spot size was set to 300 μm, and the sampling area was adjusted to 60 × 60 mm. Thermo Scientific K-Alpha point analysis assessed the relative elemental atomic ratios^14,15^. Peak fitting was performed on high resolution spectra using CasaXPS software^75^. All spectra were calibrated to the aliphatic hydrocarbon peak at 285.00 eV (labelled as C1 below). A Tougaard type (U 2 Tougaard) background and a convoluted Gaussian-Lorentzian (GL(40)) line shape were used to model the experimental data. All experimental groups were cultured on an identical Matrigel coating prepared at the same concentration, ensuring complete coverage of the growth surface. This standardized ECM background minimizes variability due to matrix-derived signals in surface-sensitive XPS analysis. Given the approximately 10 nm sampling depth of XPS and the continuous cell layer over the Matrigel, signals predominantly originate from the cell surface rather than from the underlying matrix. Therefore, any between-group differences in elemental composition are unlikely to arise from variability in the ECM layer.

### Statistical analysis

Statistical analyses were performed based on data distribution and variance homogeneity. Normality was assessed using the Shapiro-Wilk test, and homogeneity of variances was evaluated with Levene’s test. For normally distributed data, one-way ANOVA followed by Bonferroni post-hoc analysis was used. Non-normally distributed data were analyzed using the Kruskal-Wallis test with pairwise comparisons. RT-PCR data were processed using the ΔΔCt method for relative gene expression analysis, and a two-fold change was considered biologically significant. Differences in spectral data were evaluated using hierarchical clustering, principal component analysis (PCA), and Student’s t-test for each wavenumber. Elemental composition variations obtained from SEM-EDS and XPS were compared across experimental groups using appropriate statistical tests. Morphological and fluorescence intensity measurements were analyzed using ImageJ/Fiji software. Results are presented as mean ± standard deviation (SD) unless stated otherwise, and statistical significance was set at *p* < 0.05, *p* < 0.01, *p < 0.001.

## Supplementary Information

Below is the link to the electronic supplementary material.

Supplementary Material 1

## Data Availability

Data is provided within the manuscript and supplementary information files.
