# Correction: Integrated spectroscopic and morphological analyses reveal cellular shifts in gene-silenced melanoma CSCs

**DOI:** 10.1038/s41598-025-24971-z

**Published:** 2025-10-22

**Authors:** Berrin Ozdil, Günnur Güler, Evren Ataman, Huseyin Aktug

**Affiliations:** 1 Department of Histology and Embryology, Faculty of Medicine, Ege University, Izmir, 35100 Turkey; 2 Department of Histology and Embryology, Faculty of Medicine, Suleyman Demirel University, Isparta, 32260 Turkey; 3 Department of Physics, Izmir Institute of Technology, Izmir, 35430 Turkey

Correction to: *Scientific Reports* 10.1038/s41598-025-17155-2, published online 25 August 2025

The original version of this Article contained an error, where the legends of Figure 4 and Figure 5 were erroneously added in the Materials and methods section, under the subheading ‘Statistical analysis’. The duplicate legends have now been removed from the body of the text.

The original Article has been corrected.

