# Supplementary material for: Integrated spectroscopic and morphological analyses reveal cellular shifts in gene-silenced melanoma CSCs
Source: Sci Rep. 2025 Aug 25;15:31175. doi: 10.1038/s41598-025-17155-2 (PMC12379142; doi:10.1038/s41598-025-17155-2)
Supplement: Supplementary file 1 — Supplementary Material 1 [file 41598_2025_17155_MOESM1_ESM.docx]

**Supporting Information**

**SI Table 1:** SEM-EDS analysis of matrigel. Carbon, nitrogen, oxygen, sodium, sulfur, and potassium were detected in the matrigel content.

|  | C | N | O | Na | S | K |
| --- | --- | --- | --- | --- | --- | --- |
| matrigel | 6.38 | 0.01 | 61 | 22.67 | 0.16 | 9.78 |
|  | 6.1 | 0.01 | 60.9 | 22.94 | 0.19 | 9.86 |
|  | 5.69 | 0.01 | 61.2 | 23 | 0.21 | 9.88 |
|  | 5.8 | 0.01 | 61.22 | 22.85 | 0.19 | 9.92 |
|  | 5.9925 | 0.01 | 61.08 | 22.865 | 0.1875 | 9.86 |

**SI Table 2:** SEM-EDS analysis specific to CD133+ cell group.

| central | C | N | O | Na | S | K |
| --- | --- | --- | --- | --- | --- | --- |
|  | 56 | 9 | 21.97 | 5.01 | 1.06 | 6.96 |
|  | 54.31 | 10.39 | 22.44 | 5.16 | 1.02 | 6.69 |
|  | 54.94 | 11.22 | 26.56 | 3.74 | 1.13 | 2.41 |
|  | 61.02 | 5.38 | 21.91 | 2.87 | 0.84 | 7.98 |
|  | 60.22 | 2.9 | 18.26 | 2.81 | 0.7 | 15.11 |
|  | 54.1 | 12.01 | 26.54 | 3.87 | 1.12 | 2.35 |
|  | 59.49 | 6.63 | 22.27 | 3.08 | 0.85 | 7.67 |
|  | 58 | 4.82 | 19.04 | 3.12 | 0.73 | 14.29 |
|  | 52.13 | 9.26 | 28.1 | 5.68 | 0.63 | 4.2 |
|  | 50.91 | 10.39 | 28.17 | 5.83 | 0.63 | 4.07 |
|  | 58.1 | 6.92 | 24.27 | 4.48 | 1.25 | 4.98 |
|  | 57.13 | 8.37 | 25.53 | 4.39 | 1.29 | 3.29 |
|  |  |  |  |  |  |  |
| peripheral | C | N | O | Na | S | K |
|  | 49.03 | 9.95 | 25.74 | 7.08 | 0.71 | 7.49 |
|  | 47.66 | 11.17 | 26.02 | 7.24 | 0.72 | 7.19 |
|  | 37.32 | 7.66 | 36.21 | 9.98 | 0.46 | 8.38 |
|  | 35.64 | 7.48 | 37.31 | 10.28 | 0.43 | 8.85 |
|  | 36.49 | 8.84 | 36.11 | 10.05 | 0.47 | 8.04 |
|  | 34.62 | 8.62 | 37.35 | 10.42 | 0.44 | 8.55 |
|  | 33.84 | 7.33 | 40.5 | 11.31 | 0.64 | 6.37 |
|  | 31.43 | 12.2 | 41.36 | 9.85 | 0.32 | 4.84 |

**SI Table 3:** SEM-EDS analysis specific to CD133- cell group.

| central | C | N | O | Na | P | Al | S | K | Ti | Zn |
| --- | --- | --- | --- | --- | --- | --- | --- | --- | --- | --- |
|  | 47.5 | 12.18 | 29.12 | 6.22 | 0 | 0 | 0.6 | 4.37 | 0 | 0 |
|  | 35.5 | 9.83 | 40.09 | 9.34 | 0 | 0 | 0.15 | 5.09 | 0 | 0 |
|  | 43.64 | 8.94 | 33.42 | 8.46 | 0 | 0 | 0.23 | 5.31 | 0 | 0 |
|  | 29.6 | 7.56 | 45.28 | 11.25 | 0 | 0 | 0.17 | 6.13 | 0 | 0 |
|  | 28.66 | 6.82 | 47.65 | 11.11 | 0 | 0 | 0.19 | 5.56 | 0 | 0 |
|  | 54.22 | 9.7 | 27.93 | 4.57 | 0 | 0 | 0.78 | 2.81 | 0 | 0 |
|  | 34.94 | 11.97 | 38.17 | 9.14 | 0 | 0 | 0.2 | 5.59 | 0 | 0 |
|  | 45.38 | 7.45 | 26.34 | 9.62 | 0 | 0 | 7.65 | 3.56 | 0 | 0 |
|  | 42.29 | 14.39 | 34.14 | 2 | 0 | 0 | 0 | 3.54 | 1.48 | 2.16 |
|  | 50.45 | 12.42 | 23.19 | 2.41 | 0 | 2.11 | 0 | 4.48 | 1.98 | 2.94 |
|  | 47.73 | 11.09 | 33.66 | 0 | 0 | 0 | 0 | 3.74 | 1.67 | 2.12 |
|  | 47.8 | 13.53 | 31.13 | 1.53 | 0.59 | 0 | 0 | 2.81 | 1.17 | 1.44 |
|  | 52.04 | 12.98 | 24.89 | 1.83 | 0.67 | 1.6 | 0 | 3.02 | 1.28 | 1.7 |
|  | 53.33 | 10.99 | 25.81 | 1.56 | 1.17 | 1.56 | 0 | 2.79 | 1.2 | 1.58 |
|  | 50.96 | 16.82 | 28.32 | 0 | 1.2 | 1 | 0 | 1.71 | 0 | 0 |
|  | 57.74 | 0 | 35.38 | 1.39 | 0 | 0 | 0 | 2.84 | 1.21 | 1.44 |
|  | 55.07 | 10.82 | 26.63 | 0 | 1.45 | 0 | 0 | 3.01 | 1.26 | 1.76 |
|  |  |  |  |  |  |  |  |  |  |  |
| peripheral | C | N | O | Na | P | Al | S | K | Ti | Zn |
|  | 29.43 | 2.5 | 49.57 | 11.78 | 0 | 0 | 0.22 | 6.49 | 0 | 0 |
|  | 31.88 | 6.95 | 44.37 | 11.02 | 0 | 0 | 0.24 | 5.54 | 0 | 0 |
|  | 29.51 | 3.11 | 46.83 | 4.02 | 0 | 0 | 7.2 | 3.43 | 0 | 0 |
|  | 23.83 | 0.01 | 47.68 | 5.57 | 0 | 4.74 | 0 | 7.8 | 3.93 | 6.44 |
|  | 24.61 | 0.88 | 49.4 | 4.85 | 0 | 4.42 | 0 | 6.88 | 3.37 | 5.59 |
|  | 14.9 | 0 | 61.6 | 0 | 0 | 5.1 | 0 | 8.09 | 3.9 | 6.4 |
|  | 16.61 | 56.01 | 5.35 | 4.99 | 0 | 7.4 | 0 | 0 | 3.55 | 6.08 |
|  | 14.35 | 56.49 | 5.36 | 5.43 | P | 7.94 | 0 | 0 | 3.82 | 6.61 |
|  | 32.39 | 43.91 | 4.7 | 3.98 | 0 | 6.63 | 0 | 0 | 3.22 | 5.17 |
|  | 39.3 | 35.29 | 5.54 | 3.92 | 0 | 7.09 | 0 | 0 | 3.43 | 5.42 |
|  | 37.75 | 37.68 | 4.81 | 3.97 | 0 | 6.89 | 0 | 0 | 3.32 | 5.58 |

**SI Table 4:** SEM-EDS analysis specific to CD133+/nsiRNA cell group.

| central | C | N | O | Na | S | Al | P | Zr L | K | Ti | Zn |
| --- | --- | --- | --- | --- | --- | --- | --- | --- | --- | --- | --- |
|  | 54.82 | 10.25 | 28.58 | 4.13 | 0.52 | 0 | 0 | 0 | 1.7 | 0 | 0 |
|  | 54.21 | 10.92 | 28.46 | 4.23 | 0.52 | 0 | 0 | 0 | 1.66 | 0 | 0 |
|  | 47.9 | 11.29 | 30.69 | 6.13 | 0.4 | 0 | 0 | 0 | 3.59 | 0 | 0 |
|  | 49.83 | 8.06 | 27.98 | 7.58 | 0.66 | 0 | 0 | 0 | 5.89 | 0 | 0 |
|  | 47.06 | 12.1 | 30.65 | 6.28 | 0.41 | 0 | 0 | 0 | 3.49 | 0 | 0 |
|  | 48.62 | 9.21 | 28.09 | 7.72 | 0.67 | 0 | 0 | 0 | 5.69 | 0 | 0 |
|  | 53.05 | 7.51 | 29.88 | 5.88 | 0.5 | 0 | 0 | 0 | 3.18 | 0 | 0 |
|  | 53.54 | 7.73 | 28.54 | 5.7 | 0.59 | 0 | 0 | 0 | 3.91 | 0 | 0 |
|  | 52.16 | 8.39 | 29.84 | 6.01 | 0.5 | 0 | 0 | 0 | 3.1 | 0 | 0 |
|  | 52.44 | 8.75 | 28.58 | 5.83 | 0.6 | 0 | 0 | 0 | 3.8 | 0 | 0 |
|  | 54.83 | 8.45 | 26.35 | 2.35 | 0 | 1.46 | 1.25 | 0 | 2.69 | 1.1 | 1.53 |
|  | 45.05 | 17.62 | 32.19 | 0.47 | 0 | 1.07 | 0.67 | 0 | 1.59 | 0.62 | 0.73 |
|  | 50.73 | 15.89 | 26.18 | 0.8 | 0 | 1.3 | 0.97 | 0 | 2.13 | 0.89 | 1.1 |
|  | 49.17 | 13.93 | 25.5 | 4.71 | 0 | 1.18 | 0 | 2.8 | 2.02 | 0.68 | 0 |
|  | 49.42 | 16.14 | 24.88 | 4.42 | 0 | 1.11 | 0 | 1.82 | 1.59 | 0.6 | 0 |
|  | 49.73 | 7.73 | 25.99 | 5.42 | 0 | 1.78 | 0 | 3.74 | 4.02 | 1.59 | 0 |
|  | 49.65 | 15.91 | 24.86 | 4.44 | 0 | 1.11 | 0 | 1.83 | 1.6 | 0.61 | 0 |
|  | 54.02 | 8.52 | 25.04 | 2.61 | 0 | 1.85 | 1.12 | 0 | 3.39 | 1.46 | 1.99 |
|  | 47.64 | 15.18 | 27.04 | 2.01 | 0 | 1.58 | 1.03 | 0 | 2.81 | 1.15 | 1.55 |
|  | 46.62 | 8.47 | 28.63 | 3.4 | 0 | 2.43 | 0.86 | 0 | 4.52 | 2.01 | 3.06 |
|  |  |  |  |  |  |  |  |  |  |  |  |
| peripheral | C | N | O | Na | S | Al | P | Zr L | K | Ti | Zn |
|  | 27.98 | 6.2 | 48.1 | 11.3 | 0.18 | 0 | 0 | 0 | 6.23 | 0 | 0 |
|  | 27.26 | 7.27 | 47.82 | 11.43 | 0.19 | 0 | 0 | 0 | 6.04 | 0 | 0 |
|  | 38.84 | 5.86 | 41.13 | 9.28 | 0.24 | 0 | 0 | 0 | 4.64 | 0 | 0 |
|  | 38.06 | 6.9 | 40.91 | 9.37 | 0.25 | 0 | 0 | 0 | 4.5 | 0 | 0 |
|  | 32.21 | 14.38 | 40.35 | 8.52 | 0.19 | 0 | 0 | 0 | 4.35 | 0 | 0 |
|  | 28.68 | 7.01 | 45.88 | 11.02 | 0.2 | 0 | 0 | 0 | 7.19 | 0 | 0 |
|  | 34.1 | 11.69 | 39.23 | 8.88 | 0.21 | 0 | 0 | 0 | 5.89 | 0 | 0 |
|  | 31.58 | 15.06 | 40.21 | 8.7 | 0.2 | 0 | 0 | 0 | 4.25 | 0 | 0 |
|  | 28.08 | 8.1 | 45.57 | 11.09 | 0.21 | 0 | 0 | 0 | 6.93 | 0 | 0 |
|  | 33.43 | 12.6 | 39.05 | 9 | 0.22 | 0 | 0 | 0 | 5.7 | 0 | 0 |
|  | 28 | 10.96 | 43.39 | 3.09 | 0 | 3.08 | 0 | 0 | 5.09 | 2.41 | 3.98 |
|  | 31.98 | 7.65 | 36.36 | 4.35 | 0 | 3.82 | 0 | 0 | 6.85 | 3.38 | 5.61 |
|  | 36.05 | 9.04 | 29.78 | 4.53 | 0 | 3.5 | 0 | 0 | 7.4 | 3.48 | 6.22 |
|  | 26.65 | 7.88 | 43.7 | 10.65 | 0 | 3.24 | 0 | 0 | 5.22 | 2.42 | 0 |
|  | 33.81 | 10.37 | 34.71 | 9.48 | 0 | 3.05 | 0 | 0.49 | 5.48 | 2.61 | 0 |
|  | 33.68 | 4.89 | 34.7 | 11.24 | 0 | 3.97 | 0 | 0.64 | 7.27 | 3.61 | 0 |
|  | 26.85 | 7.55 | 43.72 | 10.7 | 0 | 3.25 | 0 | 0.25 | 5.25 | 2.43 | 0 |
|  | 33.97 | 10.13 | 34.72 | 9.51 | 0 | 3.06 | 0 | 0.49 | 5.5 | 2.62 | 0 |
|  | 34.15 | 4.67 | 34.59 | 11.17 | 0 | 3.94 | 0 | 0 | 7.24 | 3.6 | 0 |
|  | 28.12 | 4.62 | 44.95 | 4.41 | 0 | 3.82 | 0 | 0 | 6.15 | 2.93 | 5.01 |
|  | 33.95 | 4.71 | 37.73 | 4.87 | 0 | 3.86 | 0 | 0 | 6.71 | 3.14 | 5.02 |
|  | 27.94 | 7.59 | 42.37 | 4.25 | 0 | 3.71 | 0 | 0 | 6.24 | 2.95 | 4.96 |

**SI Table 5:** SEM-EDS analysis specific to CD133+/KLF4- cell group.

| central | C | N | O | Na | Al | P | S | K | Ti | Zn | Si |
| --- | --- | --- | --- | --- | --- | --- | --- | --- | --- | --- | --- |
|  | 41.36 | 16.92 | 32.82 | 5.78 | 0 | 0 | 0.46 | 2.67 | 0 | 0 | 0 |
|  | 40.05 | 15.26 | 33.28 | 7.47 | 0 | 0 | 0.39 | 3.57 | 0 | 0 | 0 |
|  | 49.59 | 9.99 | 29.05 | 6.47 | 0 | 0 | 0.78 | 4.12 | 0 | 0 | 0 |
|  | 47.24 | 12.86 | 29.09 | 6.02 | 0 | 0 | 0.59 | 4.2 | 0 | 0 | 0 |
|  | 47.96 | 9.02 | 24.44 | 3.8 | 2.74 | 0 | 0 | 5.44 | 2.53 | 4.07 | 0 |
|  | 45.79 | 11.18 | 22.49 | 4.67 | 2.62 | 0 | 0 | 5.93 | 2.72 | 4.6 | 0 |
|  | 48.96 | 0 | 28.16 | 4.79 | 3.37 | 0 | 0 | 6.53 | 3.12 | 5.07 | 0 |
|  | 45.43 | 13.83 | 23.43 | 3.48 | 2.34 | 0 | 0 | 5.16 | 2.36 | 3.96 | 0 |
|  | 43.84 | 4.89 | 18.2 | 5.42 | 4.67 | 0 | 0 | 9.8 | 4.92 | 8.25 | 0 |
|  | 48.25 | 9.29 | 22.95 | 3.86 | 2.65 | 0 | 0 | 5.77 | 2.73 | 4.5 | 0 |
|  | 53.12 | 3.12 | 9.82 | 2.01 | 1.45 | 0 | 0 | 4.42 | 1.97 | 2.98 | 21.11 |
|  | 54.68 | 9.18 | 19.18 | 3.68 | 2.23 | 0 | 0 | 5.34 | 2.29 | 3.41 | 0 |
|  | 48.92 | 9.43 | 26.09 | 3.11 | 2.47 | 0 | 0 | 4.67 | 2.1 | 3.21 | 0 |
|  |  |  |  |  |  |  |  |  |  |  |  |
| peripheral | C | N | O | Na | Al | P | S | K | Ti | Zn | Si |
|  | 57.57 | 5.57 | 23.41 | 5.98 | 0 | 0 | 0.52 | 6.94 | 0 | 0 | 0 |
|  | 40.05 | 15.26 | 33.28 | 7.47 | 0 | 0 | 0.39 | 3.57 | 0 | 0 | 0 |
|  | 33.76 | 5.41 | 44.06 | 10.8 | 0 | 0 | 0.25 | 5.72 | 0 | 0 | 0 |
|  | 37.86 | 9.33 | 37.39 | 9.63 | 0 | 0 | 0.32 | 5.46 | 0 | 0 | 0 |
|  | 37.83 | 13.82 | 35.28 | 8.05 | 0 | 0 | 0.3 | 4.72 | 0 | 0 | 0 |
|  | 30.02 | 6.24 | 47.26 | 10.87 | 0 | 0 | 0.26 | 5.34 | 0 | 0 | 0 |
|  | 41.84 | 9.75 | 28.69 | 4.14 | 2.9 | 0 | 0 | 5.69 | 2.55 | 4.42 | 0 |
|  | 35.81 | 14.35 | 31.29 | 3.86 | 2.66 | 0 | 0 | 5.35 | 2.48 | 4.21 | 0 |
|  | 28.5 | 3.45 | 44.77 | 4.34 | 3.9 | 0 | 0 | 6.43 | 3.15 | 5.45 | 0 |
|  | 37.82 | 3.99 | 34.08 | 4.76 | 3.81 | 0 | 0 | 6.88 | 3.39 | 5.27 | 0 |
|  | 34.14 | 6.83 | 36.18 | 4.46 | 3.59 | 0 | 0 | 6.5 | 3.08 | 5.22 | 0 |
|  | 35.7 | 6.89 | 32.82 | 5.17 | 3.83 | 0 | 0 | 6.93 | 3.19 | 5.47 | 0 |
|  | 36.6 | 6.84 | 31.88 | 4.98 | 3.62 | 1.04 | 0 | 6.7 | 3.08 | 5.26 | 0 |
|  | 26.03 | 5.94 | 44.01 | 4.73 | 3.99 | 0 | 0 | 6.61 | 3.23 | 5.45 | 0 |
|  | 37.02 | 11.81 | 32.39 | 3.84 | 2.98 | 0 | 0 | 5.5 | 2.45 | 4.01 | 0 |
|  | 33.08 | 8.66 | 38.54 | 4.04 | 3.23 | 0 | 0 | 5.62 | 2.56 | 4.27 | 0 |
|  | 27.15 | 4.56 | 45.58 | 4.55 | 3.78 | 0 | 0 | 6.27 | 2.98 | 5.13 | 0 |

**SI Table 6:** SEM-EDS analysis specific to CD133+/SHH- cell group.

| central | C | N | O | Na | Al | S | K | Ti | Zn |
| --- | --- | --- | --- | --- | --- | --- | --- | --- | --- |
|  | 47.01 | 6.03 | 31.69 | 8.48 | 0 | 0.51 | 6.28 | 0 | 0 |
|  | 45.71 | 7.35 | 31.73 | 8.63 | 0 | 0.52 | 6.06 | 0 | 0 |
|  | 52.56 | 10.93 | 29.1 | 4.5 | 0 | 0.47 | 2.44 | 0 | 0 |
|  | 51.92 | 11.63 | 28.98 | 4.62 | 0 | 0.47 | 2.38 | 0 | 0 |
|  | 56.37 | 4.17 | 24.54 | 7.04 | 0 | 0.86 | 7.01 | 0 | 0 |
|  | 40.44 | 6.51 | 37.45 | 9.71 | 0 | 0.37 | 5.51 | 0 | 0 |
|  | 46.29 | 10.04 | 31.94 | 6.83 | 0 | 0.53 | 4.36 | 0 | 0 |
|  | 55.02 | 5.32 | 24.81 | 7.2 | 0 | 0.87 | 6.78 | 0 | 0 |
|  | 39.62 | 7.53 | 37.32 | 9.81 | 0 | 0.38 | 5.34 | 0 | 0 |
|  | 45.38 | 11.01 | 31.85 | 6.99 | 0 | 0.53 | 4.23 | 0 | 0 |
|  | 49.19 | 6.85 | 29.96 | 3 | 2.4 | 0 | 4.25 | 1.82 | 2.53 |
|  | 52.37 | 9.93 | 26.24 | 1.94 | 2.17 | 0 | 3.71 | 1.63 | 2.02 |
|  | 53.38 | 7.45 | 22.47 | 3.01 | 2.57 | 0 | 5.26 | 2.42 | 3.44 |
|  | 31.27 | 6.47 | 40.55 | 3.82 | 3.6 | 0 | 6.17 | 3.09 | 5.04 |
|  | 31.05 | 12.44 | 40.23 | 3.02 | 2.62 | 0 | 4.69 | 2.25 | 3.68 |
|  |  |  |  |  |  |  |  |  |  |
| peripheral | C | N | O | Na | Al | S | K | Ti | Zn |
|  | 49.63 | 5.13 | 30.29 | 8.37 | 0 | 0.35 | 6.23 | 0 | 0 |
|  | 48.07 | 4.29 | 30.14 | 8.62 | 0 | 0.57 | 8.3 | 0 | 0 |
|  | 48.44 | 6.37 | 30.35 | 8.47 | 0 | 0.36 | 6.01 | 0 | 0 |
|  | 46.65 | 5.55 | 30.4 | 8.78 | 0 | 0.59 | 8.03 | 0 | 0 |
|  | 54.98 | 5.21 | 26.62 | 3.98 | 0 | 0.61 | 8.61 | 0 | 0 |
|  | 53.59 | 6.42 | 26.91 | 4.18 | 0 | 0.62 | 8.28 | 0 | 0 |
|  | 29.21 | 3.64 | 43.49 | 5.44 | 3.76 | 0 | 6.37 | 2.98 | 5.1 |
|  | 30.12 | 11.11 | 40.79 | 3.52 | 2.95 | 0 | 5.15 | 2.37 | 3.99 |
|  | 29.09 | 6.04 | 47.62 | 0 | 3.57 | 0 | 6.17 | 2.82 | 4.68 |
|  | 34.42 | 5.37 | 35.44 | 4.78 | 3.96 | 0 | 6.96 | 3.39 | 5.68 |
|  | 32.65 | 7.64 | 40.81 | 0 | 3.54 | 0 | 6.74 | 3.26 | 5.36 |
|  | 30.42 | 6.16 | 40.96 | 4.17 | 3.69 | 0 | 6.39 | 3.04 | 5.18 |
|  | 23.92 | 0 | 50.05 | 5.01 | 4.45 | 0 | 7.12 | 3.41 | 6.03 |
|  | 25.88 | 0 | 51.53 | 4.71 | 3.77 | 0 | 6.3 | 2.99 | 4.81 |
|  | 29.88 | 0 | 45.95 | 5.03 | 4.07 | 0 | 6.8 | 3.15 | 5.12 |
|  | 31.18 | 3.15 | 37.22 | 5.33 | 4.68 | 0 | 7.91 | 3.93 | 6.61 |
|  | 24 | 5.86 | 50.76 | 3.66 | 3.41 | 0 | 5.35 | 2.6 | 4.36 |
|  | 24.86 | 4 | 54.4 | 0 | 3.52 | 0 | 5.86 | 2.73 | 4.62 |
|  | 27.78 | 6.11 | 47.09 | 3.42 | 3.35 | 0 | 5.42 | 2.53 | 4.31 |

**SI Table 7:** SEM-EDS analysis specific to CD133+/HIF1a- cell group.

| central | C | N | O | Na | S | Al | K | Ti | Zn | Si |
| --- | --- | --- | --- | --- | --- | --- | --- | --- | --- | --- |
|  | 52.32 | 11.44 | 30.09 | 4.13 | 0.68 | 0 | 1.34 | 0 | 0 | 0 |
|  | 47.28 | 12.86 | 30.43 | 6.07 | 0.45 | 0 | 2.91 | 0 | 0 | 0 |
|  | 54.57 | 4.63 | 22.83 | 6.92 | 0.71 | 0 | 10.35 | 0 | 0 | 0 |
|  | 55.18 | 8.12 | 24.73 | 5.58 | 0.63 | 0 | 5.76 | 0 | 0 | 0 |
|  | 48.15 | 6.95 | 30.68 | 8.01 | 0.48 | 0 | 5.74 | 0 | 0 | 0 |
|  | 52.21 | 11.24 | 27.05 | 5.29 | 0.51 | 0 | 3.71 | 0 | 0 | 0 |
|  | 49.16 | 5.91 | 26.92 | 8.1 | 0.13 | 0 | 9.78 | 0 | 0 | 0 |
|  | 50.56 | 15.38 | 25.89 | 4.51 | 0 | 1.42 | 2.24 | 0 | 0 | 0 |
|  | 52.3 | 15.91 | 25.82 | 3.55 | 0 | 1.03 | 1.39 | 0 | 0 | 0 |
|  | 51.48 | 15.93 | 27.73 | 0 | 0 | 1.39 | 2.31 | 0 | 1.16 | 0 |
|  | 62.72 | 8.04 | 21.66 | 2.51 | 0 | 1.53 | 3.54 | 0 | 0 | 0 |
|  | 55.38 | 13.39 | 24.41 | 2.36 | 0 | 1.3 | 3.16 | 0 | 0 | 0 |
|  | 51.36 | 12.13 | 27.05 | 4.67 | 0 | 1.62 | 3.16 | 0 | 0 | 0 |
|  | 60.89 | 8.53 | 25.93 | 3.85 | 0 | 0 | 0.81 | 0 | 0 | 0 |
|  | 60.06 | 8.77 | 25.99 | 3.68 | 0 | 0 | 1.5 | 0 | 0 | 0 |
|  | 58.25 | 12.93 | 24.01 | 3.67 | 0 | 0 | 1.13 | 0 | 0 | 0 |
|  |  |  |  |  |  |  |  |  |  |  |
| peripheral | C | N | O | Na | S | Al | K | Ti | Zn | Si |
|  | 30.41 | 3.34 | 47.24 | 11.88 | 0.26 | 0 | 6.87 | 0 | 0 | 0 |
|  | 52.41 | 11.45 | 30.02 | 4.11 | 0.68 | 0 | 1.33 | 0 | 0 | 0 |
|  | 39.84 | 4.69 | 40.19 | 9.62 | 0.18 | 0 | 5.49 | 0 | 0 | 0 |
|  | 22.78 | 0 | 56.57 | 0 | 0 | 4.25 | 7.07 | 3.53 | 5.81 | 0 |
|  | 26.08 | 0 | 50.7 | 4.75 | 0 | 4.01 | 6.42 | 3.03 | 5.02 | 0 |
|  | 27.69 | 0 | 52.97 | 3.58 | 0 | 3.43 | 5.51 | 2.56 | 4.25 | 0 |
|  | 32.72 | 4.63 | 42.14 |  | 0 | 3.91 | 7.33 | 3.49 | 5.79 | 0 |
|  | 37.78 | 9.08 | 35.23 | 3.09 | 0 | 2.96 | 5.29 | 2.54 | 4.03 | 0 |
|  | 41.32 | 0 | 21.16 | 2.55 | 0 | 1.75 | 4.47 | 1.98 | 3.45 | 23.31 |
|  | 37.79 | 6.81 | 32.05 | 4.67 | 0 | 3.46 | 6.7 | 3.21 | 5.31 | 0 |
|  | 29 | 6.8 | 47.56 | 0 | 0 | 3.31 | 5.91 | 2.75 | 4.67 | 0 |
|  | 33.91 | 9.08 | 38 | 2.32 | 0 | 3.17 | 5.99 | 2.83 | 4.71 | 0 |
|  | 30.7 | 0.01 | 46.28 | 3.85 | 0 | 3.88 | 6.54 | 3.04 | 5.7 | 0 |
|  | 28.81 | 6.6 | 47.76 | 0 | 0 | 3.36 | 5.97 | 2.78 | 4.72 | 0 |


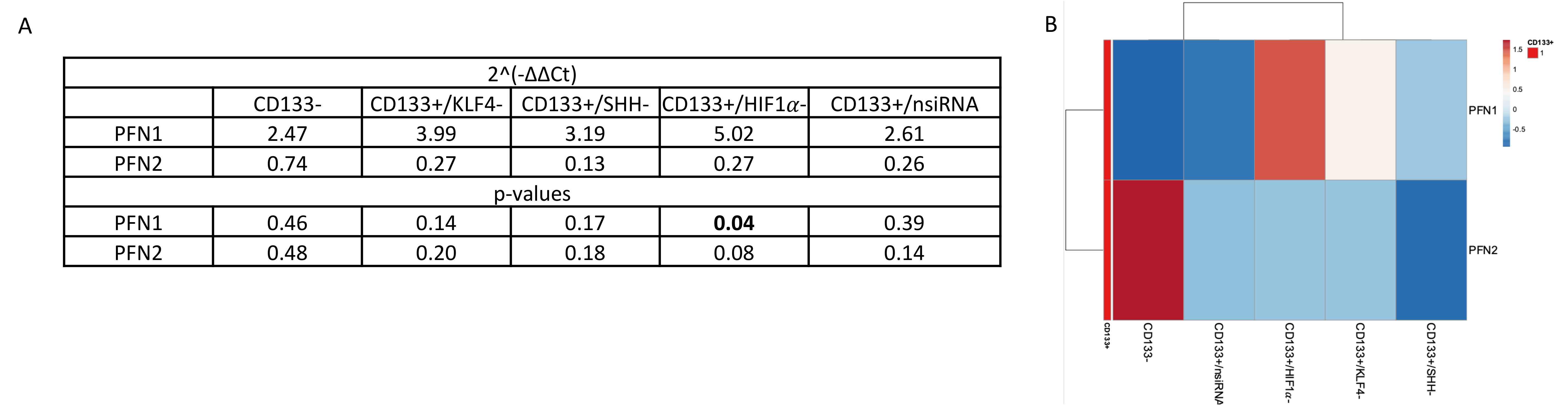


SI Fig. 1: Gene expression of PFN1 and PFN2 as normalized with CD133+ cells and gene expression fold changes were listed (A) and (B) the heatmap with hierarchical trees was generated by Clustvis.


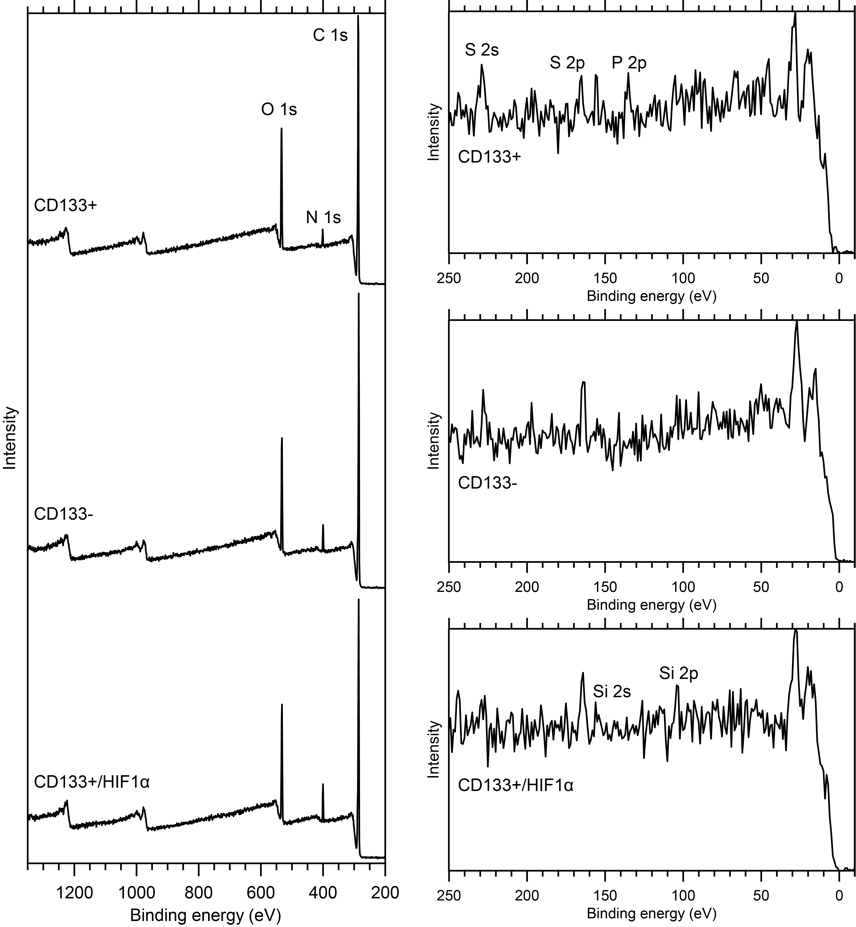


SI Fig. 2: Survey scan of XPS analysis with a binding energy range of 1350 to -10 eV for CD133+, CD133- and CD133+/HIF1α- groups.

SI Fig. 3: Survey scan of XPS analysis with a binding energy range of 1350 to -10 eV for CD133+/KLF4-, CD133+/SHH- and CD133+/nsiRNA groups.


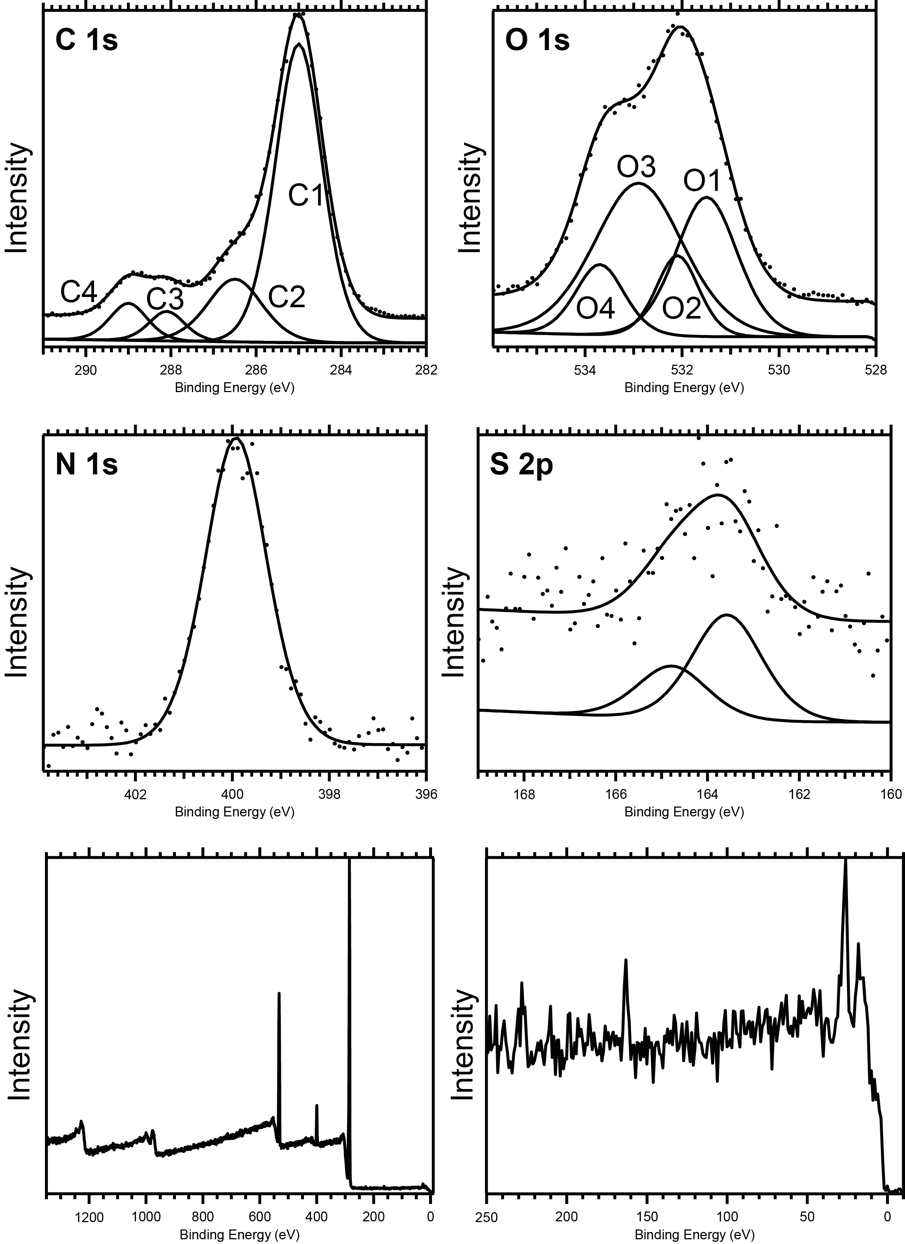


SI Fig. 4: Survey scan of XPS analysis with a binding energy range of 1350 to -10 eV for Matrigel.
